# Chikungunya Virus Diagnosis: A Review of Current Antigen Detection Methods

**DOI:** 10.3390/tropicalmed8070365

**Published:** 2023-07-17

**Authors:** Fredy Brice Nemg Simo, Felicity Jane Burt, Nigel Aminake Makoah

**Affiliations:** 1 Division of Virology, Faculty of Health Sciences, University of The Free State, Bloemfontein 9301, Free State, South Africa; burtfj@ufs.ac.za; 2 Division of Virology, National Health Laboratory Service, Bloemfontein 9301, Free State, South Africa

**Keywords:** chikungunya virus, diagnosis, chikungunya antigen detection, lateral flow assay, enzyme linked immunosorbent assay

## Abstract

Chikungunya is a mosquito-borne viral disease caused by the chikungunya virus (CHIKV). CHIKV is expanding at an alarming rate, potentially spreading and establishing endemicity in new areas where competent vectors are present. The dramatic spread of CHIKV in recent years highlights the urgent need to take precautionary measures and investigate options for control. It is crucial in developing nations where diagnostic tools are limited, and symptoms are similar to other prevalent diseases such as malaria and dengue. The most reliable method for diagnosing chikungunya virus is viral gene detection by RT-PCR. Alternative methods like detecting human antibody and viral antigen can also be used, especially in areas where resources are limited. In this review, we summarize the limited data on antigen detection immunoassays. We further explain the essential structural elements of the virus to help comprehend the scientific concepts underlying the testing methods, as well as future methods and diagnostic approaches under investigation.

## 1. Introduction

Chikungunya fever is a reemerging debilitating mosquito-borne disease characterized by severe arthritis [1]. Clinical symptoms of this disease include skin rash, myalgia, and arthralgia [2]. This disease is caused by the chikungunya virus (CHIKV), an arbovirus primarily transmitted by mosquitoes of the genus *Aedes* [3]. This virus was discovered during an outbreak in 1952 on the Makonde highland (Tanzania) and named after the Makonde word: “kungunyala”, which means: “bends up”, referring to the posture of patients suffering from severe joint pain during CHIKV infection. It is only after the major epidemic of chikungunya, which spread from the coastal towns of Kenya to the Indian Ocean Islands in 2005 to 2006, including the island of Reunion affecting nearly 40% of the population, that studies were conducted in several countries to establish the prevalence of this pathogen [4,5]. Consequently, a systematic review and meta-analysis of seroprevalence studies using pooled immunoglobulin M (IgM) and immunoglobulin G (IgG) seroprevalence in people residing in Africa indicated a prevalence outside outbreak periods of 9.7% (95% CI 3.0–19.6; 16 studies) and 16.4% (95% CI 9.1–25.2; 23 studies), respectively [6]

**Figure 1:**
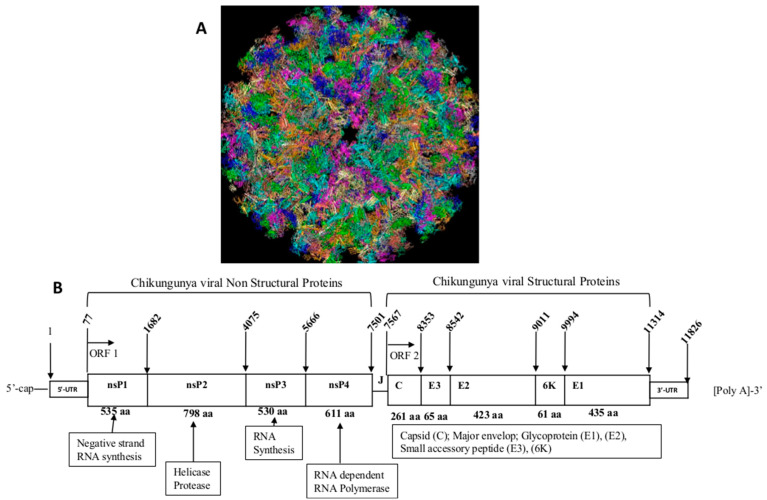
(**A**) Morphological structure of the virus adapted from [10], (**B**) structural and nonstructural proteins of the virus. Legend: ORF (Opened reading frame; nsP (Nonstructural proteins; C (Capsid); E (Envelop) J (Junction).

CHIKV is an approximately 12-kb positive-sense RNA (+ssRNA) genome containing a 5′-methylguanylate cap, a 3′-polyadenylated tail, and two ORFs. It is a member of the family *Togaviridae*, genus *Alphavirus*. The *Togaviridae* family of viruses belong to group IV of the RNA viruses. The genus Alphavirus has a wide range of viruses and the medically relevant are: Sindbis (SINDV), Eastern Equine Encephalitis (EEEV), Semliki Forest virus (SFV), Western Equine Encephalitis (WEEV), Ross River (RRV) and o’nyong-nyong (ONNV) [7]. CHIKV is a spherical virus of 70 nm in diameter. It consists of a protein capsid and a lipoprotein envelope from the host. The genome of CHIKV encodes for four non-structural proteins (nsP1-4), with five structural proteins (C, E3, E2, 6K and E1) expressed from subgenomic RNA synthesized in infected cells [4]. Figure 1 illustrates the morphology and complete genome of CHIKV [7,8,9]. 

The structural proteins of CHIKV are synthesized by translation of subgenomic RNA located on the second 3′UTR-ORF. This region is translated to one polyprotein which is post-translationally cleaved to form five proteins. Glycoproteins E1 and E2 play an important role in the pathogenicity of the virus as they are involved during the fusion of the virus to the host cell (E1) and interaction with host receptors (E2) before fusion [11]. The crystal structures of the precursor p62–E1 heterodimer and mature E3–E2–E1 glycoprotein complexes have been reported [11]. Cleavage of the alphavirus precursor glycoprotein p62 into the E2 and E3 glycoproteins before assembly with the nucleocapsid has been shown to be the key to producing fusion-competent mature spikes on alphaviruses including CHIKV [12]. Expression of full-length CHIKV 6KE1 and E3E2 glycoproteins using baculovirus vectors in insect cells showed glycosylation, furin processing, plasma membrane translocation of E1 and E2, and retention of CHIKV-E1 functional activity as a membrane fusion protein [13]. Many other studies have revealed important information regarding the CHIKV genome and structure, which has helped further development of new diagnosis tools, antiviral treatment, and vaccine development.

## 2. Physiopathology of Chikungunya Virus Infection

The main vectors of CHIKV are *Aedes aegypti.* Although *Aedes aegypti* is the classical vector for CHIKV, the 2005 outbreak in La Réunion was associated with an atypical vector, *Aedes albopictus*. The geographic and temporal dispersion of the insect vectors, their growth rate, and the length of the viral incubation period inside them are only a few of the variables that affect how successfully arboviral illnesses are transmitted. *Aedes albopictus* is currently found in sub-Saharan Africa, several European nations, and around 25% of the United States [14]. Because it is aggressive, silent, and diurnal, *Aedes albopictus* can successfully infect both humans and animals. It is thought to have started off as a zoophile before evolving into an anthropophile. The E1 Ala226Val mutation and other mutations that have recently been found in E2 also govern CHIKV adaptation to its mosquito hosts. These changes were seen in E1, a class II viral fusion protein that mediates viral entry at low pH [15]. The virus is primarily transmitted by mosquito bite; however, maternal-fetal transmission has been reported [15]. The virus reproduces inside the midgut of a mosquito and spreads to auxiliary tissues like the salivary glands. Compared to other mosquito-borne viruses, CHIKV can infect a new, naive host more quickly; laboratory tests have shown that the virus can be found in the mosquito saliva as soon as 2–3 days after the blood meal [16]. Therefore, the transmission cycle between humans and mosquitoes and vice versa can occur in less than a week. The mosquito is thought to be able to transmit the virus for the remainder of its life once it becomes infected. In mammals, CHIKV replicates in the skin and fibroblasts, enters the bloodstream, and disseminates to the liver, muscles, joints, lymphoid tissues, and brain [17]. CHIKV activates non-hematopoietic cells, especially fibroblasts, which in turn indirectly induces the synthesis of type I interferon (IFN), an action that is crucial for removing CHIKV from the body. Neonates lacking either interferon regulatory factor (IRF) were noticeably more susceptible to infection, even though IRFs 3 and 7 operated redundantly in adults [18].

Additionally, CHIKV seems to activate the interferon promoter stimulator 1 (IPS-1), which results in the accumulation of IRF3-dependent mRNAs and prevents these mRNAs from encoding proteins. Acute patients’ cutaneous rashes contain CD8+ lymphocytes, but chronic patients’ synovial effusions mostly contain CD4+ T cells. Bone marrow stromal antigen 2 (BST-2) may guard lymphatic tissues and control inflammatory reactions brought on by CHIKV in the host. A sudden onset of fever, typically accompanied by joint pain, is a defining feature of chikungunya. The joint discomfort, which often lasts for a few days but can last for weeks, months, or even years, is frequently excruciating. The virus can therefore lead to acute, subacute, or chronic illness. Muscle soreness, joint swelling, headaches, nausea, exhaustion, and rashes are other typical symptoms [19]. Unlike dengue, chikungunya rarely worsens to the point where it threatens life. Occasionally cases of cardiac, neurological, and ophthalmological problems and gastric discomfort have been reported, although serious consequences are uncommon. However, in elderly patients with underlying illnesses, the disease can be a factor in death. Although most people fully recover from the infection, joint pain can occasionally last for months or even years [20]. Thus, the necessity to prevent or treat infections as soon as they are diagnosed.

## 3. Diagnosis of Chikungunya Virus Infection

**Figure 2:**
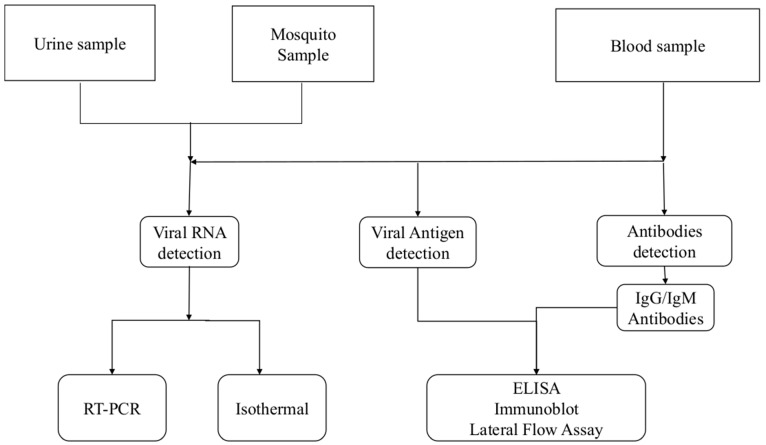
Algorithm of the diagnosis of chikungunya virus infections.

Laboratory diagnosis is key to reduce the morbidity caused by CHIKV infection. Currently, several methods have been developed commercially or in-house for the detection of CHIKV [21]. The laboratory diagnosis of CHIKV depends on the kinetics of markers of viral infection (Figure 2). Based on this, different diagnostic algorithms are designed to detect specific anti-CHIKV IgG/IgM antibodies; viral specific antigens and/or viral genome.

Molecular techniques such as RT-PCR and isothermal amplification are used for the detection of the CHIKV genome. The viral antigen detection is achieved by using an enzyme linked immunosorbent assay (ELISA), an immunofluorescence assay (IFA), a rapid diagnostic test (RDT), and immunoblotting methods. Plate reduction neutralization tests (PRNT) remain the gold standard for serology and confirm the presence of neutralizing antibodies but are seldom used routinely for diagnosis. This technique requires trained personnel working under specified experimental and biological safety level (BSL) conditions [21,22,23].

The diagnosis of CHIKV remains a challenge because its clinical manifestations are not specific and difficult to differentiate from other infections occurring in similar geographic regions, including malaria, typhoid, and dengue; which share common symptoms with CHIKV infection [24]. The current gold standard method for CHIKV detection is RT-PCR, but this method requires well-trained personnel, sophisticated equipment, and a laboratory. This method remains expensive for developing countries where this virus is endemic or re-emerging [25]. For this reason, the diagnosis of CHIKV is under-estimated in developing countries compared to developed countries [21,26]. Serological diagnostics are mostly used in developing countries because they are less expensive and do not require highly trained personnel to process samples. However, these serological techniques detecting specific anti-CHIKV IgG and IgM may be non-specific with possibilities of cross reactivity between the CHIKV antibody and other alphaviruses due to the similarity between the structure of their tri-dimensional epitope, allowing the antibody for one to identify the antigen of the other one. These methods then require a confirmation using the gold standard assay, which requires detection of neutralizing antibodies by the plate reduction neutralization test or microneutralization assays, which requires a sophisticated high biosafety laboratory setting and welltrained personnel [27]. Antigen detection may be a suitable approach for rapid and easy detection of CHIKV because its presence is directly linked to the presence of the virus. The landscape of CHIKV RDT is fragmented and needs coordinated efforts to ensure that patients in CHIKV-endemic areas have access to appropriate RDT. Further research is crucial to determine the impact of such tests on integrated fever case management and management practices for acute febrile patients [25].

## 4. Chikungunya Detection Using Viral Antigen E1 and E2 as a Target

Three types of tests are generally used for CHIKV antigen detection, ELISA, IFA, and RDT. ELISA is among the most common tests used in developing as well as developed countries. The first ELISA were developed to reduce the use of radioactive elements for revelation and create a fast, simple, and safe alternative [28]. The analytes detected using an ELISA are usually proteins, and sample types can range from raw biological fluids (e.g., plasma, serum, urine, sweat) to refined cell culture media or purified recombinant proteins in solution [29]. IFA is an immunolabeling technique that uses antibodies and fluorochromes. For CHIKV diagnosis, the IFA reveals a specific protein (non-structural or structural proteins) directly in the cell, by fluorescence emission. It allows to the determination of not only the presence or absence of a protein, but also its localization in the cell or the analyzed tissue [30]. The RDT is used to quickly establish the presence or level of analyte for diagnostic purposes, such as a disease marker (antibodies, antigens) or physiological parameter. RDT usually involve chemical or enzymatic or immunological reactions, which produce a specific coloration that allows for immediate interpretation of the result. They are designed to be used in the doctor’s office but also at the patient’s bedside (point of care), or for certain applications by the individual himself, and in the field (field tests) for the environment, or finally for contamination control in industry [31]. Thus, it is important to reveal the results within a few minutes.

We review a limited number of articles describing the development and validation of CHIKV assays for the detection of antigens (Table 1).

Various approaches to the development of CHIKV antigen detection have previously been described. A study by Kashyap et al. (2010) described a CHIKV diagnosis in an Indian population using an indirect ELISA for antigen detection. Antigens were prepared using an Indian strain of CHIKV, ISW HYD06 (GenBank accession number 876190), which belongs to the ECSA genotype and was previously isolated from a patient from Hyderabad, India, during the 2006 epidemic [32]. Chikungunya antigens standardization was done by titration using IgG ELISA, and an antigen detection ELISA that can screen serum from patients clinically suspected of having CHIKV infection was used. Antigen was detected in most of the CHIKV-infected patients. The sensitivity of antigen detection was 85%, which was significantly higher (*p* < 0.001) compared with IgM and IgG ELISAs. Antigen detection ELISA gave a positive confirmatory result in the early phase of the disease, and was also valuable in the subclinical stage and may be useful for field applications for the rapid detection of CHIKV infection [32]. This ability to detect infection early is due to the initial burst of virus replication and is associated with high levels of viremia, during which time the individual is highly infectious. The authors observed that the rate of positive antigen detection was highest (95%) in the acute phase (first 5 days) and then gradually diminished, which is an important finding, because it indicates the possibility of a definitive diagnosis of this viral disease being obtained at a very early stage. 

Ref. [33] described an antigen detection based immunochromatographic (IC) test using monoclonal antibodies produced after immunization of mice with a clinical isolate of the CHIKV ECSA genotype [33]. The hybridomas producing antibodies were screened using an immunofluorescence assay (IFA) on CHIKV Thai isolate-infected Vero cells. A rapid diagnostic test was then developed using the characterized monoclonal antibodies, which reacted to ECSA, Asian, and West African genotypes of CHIKV [33]. The authors examined 112 samples from four countries using RT-PCR and IgM ELISA and positive samples were then tested them using the developed IC test and found it produced 68 true positive (60.7%), 34 true-negative (30.3%), 2 false-positive (1.8%), and 8 false-negative (7.1%); and a sensitivity and specificity of 89.4% and 94.4% respectively, and concordance of 91.1% compared to PCR. 

Ref. [34] evaluated an IC rapid diagnosis kit for detection of chikungunya virus antigen in India. The authors examined Sera from 104 qRT-PCR CHIKV-positive and/or IgM-positive (ELISA) subjects collected in 2016; 15 samples from CHIKV-negative/DENV-positive and 4 samples from healthy individuals [34]. The authors found that by using qRT-PCR as the gold standard, the IC assay’s sensitivity, specificity, and overall agreement were 93.7, 95.5, and 94.3%, respectively. Additionally, there was a significant positive association between the IC kit score and CHIKV RNA copy number. The IC kit did not cross-react with DENV NS1-positive/CHIKV-negative samples and was able to identify CHIKV antigen in patient sera that were also infected with DENV. The results from this study showed that the IC kit may be useful in areas where the CHIKV ECSA genotype is endemic and have high probability to occur as co-infections with dengue. The main limitation of this study was that the IC kit was not tested on other genotype of CHIKV.

Ref. [35] used mouse monoclonal antibodies as a tracer against the E1-envelope protein of chikungunya to evaluate an IC rapid test. When tested in a reference setting, the diagnostic sensitivity of E1 was 88.9% for the ECSA genotype but only 33.3% for the Asian genotype. The overall diagnostic specificity was 76.9% in the CHIKV-negative endemic panel, a value considered moderate and warranting further research to improve the specificity. The data further suggested that the current format using the E1 antigen will not be suitable for their diagnosis. To improve the assay, Suzuki et al. (2020) prepared a new monoclonal antibody with the aim of improving the sensitivity to the Asian genotype by using E1 pseudolentiviral vectors as antigen, to develop a novel IC RDT. They observed an increase in sensitivity, up to 100% for reactivity with the ECSA genotype but found no improvement of sensitivity and specificity for the Asian genotype. To increase the sensitivity and specificity to the Asian genotype, a rapid LFA and ELISA were developed and validated using a combination of E1/E2 antigens [37]. Samples from a cohort of 100 patients were used and a sensitivity of 51% was obtained, while the specificity was 96.67% for ELISA. A sensitivity and specificity of 100% were obtained with LFA. These results were found to be the most promising for chikungunya diagnosis.

In a study aiming at overcoming the limitations of molecular diagnostics in asymptomatic individuals, ref. [36] developed two novel monoclonal antibodies targeting CHIKV E1 and applied in a fluorescence-linked immunosorbent assay (FLISA) using coumarin-derived dendrimer as the fluorophore. The method detects CHIKV 3-fold better than ELISA in human sera and blood. They observed that sera and blood appeared to interfere with ELISA. However, the sensitivity and specificity of this assay was not evaluated [36].

Ref. [38] generated CHIKV antibodies by using two combinations: Combination A (48 and 155) generated previously in mice; and Combination B produced by immunizing a C57BL/6 mouse with CHIKV virus-like particles (Native Antigen CHIKV-VLP Q5XXP3.1) [38]. They found that the two antibody pairs, Combination A and B, were chosen based upon the high CHIKV virus-like particle (VLP) binding affinity and discrimination between the CHIKV VLP and Mayaro virus (MAYV) VLP determined from the ELISA and flow cytometry of infected cell screening, as well as the low limits of detection. The authors collected 1056 antibodies from the hybridoma and measured the ability of antibodies to bind to the VLP by ELISA. The authors evaluated the antibody pairs on a dipstick format and selected based upon the lowest limit of detection and dissociation constant through image analysis. The E1/E2 CHIKV ELISA was validated using 100 PCR-confirmed Chikungunya samples and 60 negative samples from Honduras. The results revealed that the performance of the ELISA ranged from 41.33% to 95.45% in sensitivity and 84.76% to 98.02% in specificity over the Ct range of 20 to 35. The sensitivity and specificity reached 100% for both lateral flow tests using 39 samples from Colombia and Honduras at Ct cutoffs of 20 and 27, respectively. For both lateral flow tests, sensitivity decreased as the Ct increased after 27. Because CHIKV E1/E2 are exposed in the virion surfaces in serum during the acute infection phase, these sensitive and specific assays demonstrate opportunities for early detection of this emerging human pathogen.

Cross-reactions concern viruses of neighboring species and belonging to the same species; they are easily explained, because a virus is not a single antigen, but a real “mosaic of antigens”. When an animal is immunized by injecting whole particles, it reacts by producing multiple antibodies directed against all the injected antigens. If two viruses have an identical antigen in their mosaic, the immune sera obtained in the animal will give a cross-reaction between these two viruses [39]. This phenomenon is observed, for example, in alphaviruses in general including the CHIKV, SINDV as an example. The serum of a patient with chikungunya fever will therefore agglutinate not only SINV but also ONNV, making the interpretation of the serodiagnosis of the disease sometimes delicate. The existence of cross-reactions between two antigens which are really different, but which have identical or similar antigenic determinants on their molecule, has been demonstrated; the weaker the relationship between these determinant sites, the weaker the cross-reaction [40]. The PRNT, microneutralization methods are well suited to the study of the structural kinship that can exist between various antigens and allow today the identification of the specificities of antibodies [41].

When developing these assays, researchers frequently focus on virus strains that are prevalent in their geographic area and ignore strains from other regions or continents. Future research should include additional related alphaviruses like the Ross River virus (RRV), which is prevalent in Australia and the Pacific Island regions, and the ONNV, which is found in Sub-Saharan Africa. Future research should examine how well assays perform with inactivated serum samples so that non-infectious material can be used for point-of-care testing, especially in regions with low resources.

## 5. Future Perspectives on Chikungunya Diagnostics

CHIKV is expanding at an alarming rate with the potential to spread and establish endemicity in new areas where competent vectors are present. The dramatic spread of CHIKV in recent years highlights the urgent need to take precautionary measures and investigate options for control. This is particularly important in developing nations where diagnostic tools are limited, and symptoms are like those of other diseases such as malaria and other medically relevant alphaviruses. The E1 and E2 glycoproteins are mainly responsible for membrane fusion and virus entry into host cells, where E2 interacts with the cellular receptor and functions in attachment to cells, and E1 participates in virus fusion to the cell membrane [42]. Hence, the glycoproteins are frequent targets for assay development. The prevention and control of CHIKV infection necessitate improving current CHIKV diagnostic approaches to detect early and low CHIKV antigen levels and correctly distinguish CHIKV infections from those due to other medically relevant alphaviruses, and most importantly to other febrile infections. We searched the national center for biotechnology information (NCBI), with the terms “alphavirus, E1” and “alphavirus, E2”. We then selected the UniPROTKB/Swiss-Prot database as it is considered a high quality manually annotated and non-redundant protein sequence database. Sequences were aligned using the E1 and E2 of the Chikungunya virus strain S27-African prototype (Q8JUX5.3) as a reference. Sequences were aligned using Mega 11 and all incomplete sequences were removed from the analysis. We then used Mview to visualize the alignments (Files S1 and S2).

The similarities between CHIKV and ONNV are higher than 90% for E1 and E2, and between 30–60% for other medically relevant alphaviruses (S1 and S2). From these observations, it is understandable that differentiating CHIKV and ONNV using antigen detection techniques will be challenging; however, a careful analysis of a non-conserved region could help differentiate CHIKV/ONNV from other viruses. This can be achieved by conducting structural studies of the target antigens and epitope mapping using X-ray co-crystallography and cryogenic microscopy (Cryo-EM), array-based oligo-peptide scanning, or site-directed mutagenesis. E1 and E2 are the main targets in the development of diagnosis. However, recent studies have been focusing on E2 most probably because of its lower similarities compared with E1 or simply because it is a better immunogen. The suitability of E2 as the preferred antigen is further highlighted in the study of Suzuki et al. (2020) where an increase of sensitivity and specificity was obtained after E1 was combined with E2 [29]. The recombinant E1 and E2 envelope proteins have been successfully expressed in insects cells [39,43], in E. coli [44,45,46], and HEK-293 cells [44], and used to develop a serologic assay to identify IgG and IgM. However, assays that detect the virus antigens will further help in identifying active infection and help start treatment earlier and as a result, curb potential outbreak.

Few studies have reported the development of monoclonal antibodies. Increasing the number of monoclonal antibodies isolated from humans and other species could help develop a more sensitive and specific serological assay. Three distinct domains have been identified in the E2 protein: A (16–134 aa), B (173–231 aa), and C (269–341 aa), which are involved in receptor binding and considered immunogenic [12,23,47]. Additionally, the N218 of CHIKV E2 was identified as a potent neutralizing epitope as it mediated the binding and neutralization of the monoclonal antibody 3E7b [48]; because N218 is only observed on the CHIKV and ONNV E2, this antibody could be used to distinguish between these two closely related viruses and the other relevant alphaviruses. Other epitopes, including linear epitopes, have been identified in E2 and monoclonal antibodies (mAbs) against CHIKV E2 were produced and their sensitivity and specificity were compared with commercially available mAbs, using ELISA [49]. Peptides E2P3, E2P7, E2P16, and E2P17 were revealed as the most immunodominant peptides that together can form the basis for designing an accurate, economical, and easy to synthesize peptide-based immunodiagnostic for CHIKV [50,51].

## 6. Conclusions

The COVID-19 pandemic has proven the worth of rapid diagnostics in detecting emerging infectious diseases. The need for accurate RDT for infectious diseases has been highlighted by many and they are currently under development. The lateral flow assay developed by [38] presented the best characteristics when compared with polymerase chain reaction. But this assay showed high specificity with samples that have a Ct value lower than 28; it is therefore a good assay for the early diagnostic of chikungunya infection. However, the evaluation of these assays will need to be harmonized to ensure comparability among studies and diagnostic accuracy. Only a small portion of the world’s population has been tested, and the priority has understandably been given to those who exhibit the disease’s severe clinical symptoms and frontline healthcare providers. This is despite the unprecedented collective effort made by the researchers to develop sensitive, selective, and rapid test kits. Until we discover efficient and scientifically verified treatments and vaccines, the battle against the chikungunya virus could be protracted. Until then, researchers and diagnostic tools manufacturers should concentrate on developing affordable, user friendly, and rapid tests to track the true scope and progression of the disease globally to enable policymakers to take appropriate action.

## Figures and Tables

**Table 1:** Chikungunya developed kits for the detection of virus antigens.

Method	Target of the Assay	Sensitivity	Specificity	Reference
ELISA	Chikungunya viral antigen	85%	/	[32]
Immuno-chromatography	Chikungunya viral antigen	89.4%	94.4%	[33]
Immuno-chromatography	E	93.7%	95.5%	[34]
Immuno-chromatography	E1	8/9 (88.9%, 95% CI 56.5–98.0) ECSA genotype, 10/30 (33.3%, 95% CI 19.2–51.2)	49/59 (83.1%, 95% CI 71.5–90.5)	[35]
FLISA	E1	/	/	[36]
Immuno-chromatography	E1	92% (92/100) (95% CI 85.0–95.9)	100% (100/100)	[37]
Lateral flow assay/ELISA	E1/E2	51% (ELISA) and 100% (LFA)	96.67% (ELISA) and 100% (LFA)	[38]

ECSA: East Central South African genotype; LFA: Lateral Flow Assay; FLISA: Fluorescence Linked Immunosorbent Assay; CI: Confidence Interval.

## Supplementary Materials

The following supporting information can be downloaded at: https://www.mdpi.com/article/10.3390/tropicalmed8070365/s1, File S1: E1 Alignment; File S2: E2 Alignment.
