# Supplementary material for: Chikungunya Virus Diagnosis: A Review of Current Antigen Detection Methods
Source: Trop Med Infect Dis. 2023 Jul 17;8(7):365. doi: 10.3390/tropicalmed8070365 (PMC10383795; doi:10.3390/tropicalmed8070365)
Supplement: Supplementary file 1 [file tropicalmed-08-00365-s001.zip › MView-E1 alignment.pdf]

```
Reference sequence (1): sp|Q8JUX5.3|POLS
Identities normalised by aligned length.
Colored by: identity

                              cov     pid   1 [         .         .         .         .         :         .         .         . 80
 1 sp|Q8JUX5.3|POLS 100.0% 100.0%     RTAKAAT-YQEAAVYLWNEQQPLFWLQALIPLAALIVLCNCLRLLPCCCKTLA------FLAVMSIGAHTVSAYEHVTVI
 2 sp|Q5WQY5.1|POLS 100.0%  96.8%     RTAKAAT-YQEAAVYLWNEQQPLFWLQAIIPLAALIVLCNCLRLLPCCCKTLT------FLAVMSVGAHTVSAYEHVTVI
 3 sp|Q5XXP3.1|POLS 100.0%  96.4%     RTTKAAT-YYEAAAYLWNEQQPLFWLQALIPLAALIVLCNCLKLLPCCCKTLA------FLAVMSIGAHTVSAYEHVTVI
 4 sp|P22056.1|POLS 100.0%  87.3%     RTAKAAS-YYEAATYLWNEQQPLFWLQLLIPLSAAIVVCNCLKLLPCCCKTLT------FLAVMSIGARTVTAYEHATVI
 5 sp|O90371.1|POLS 100.0%  88.1%     KTAKAAS-YYEAATYLWNEQQPLFWLQLLIPLSAAIVVCNCLKLLPCCCKTLT------FLAVMSIGARTVSAYEHATVI
 6 sp|O90369.1|POLS 100.0%  87.9%     KTAKAAS-YYEAATYLWNEQQPLFWLQLLIPLSAAIVACNCLKLLPCCCKTLT------FLAVMSIGARTVSAYEHATVI
 7 sp|Q86925.2|POLS  98.4%  44.6%     QRTSADE-FTDTMGYLWQHSQTMFWIQLVIPLAAVITLVR---CCSCCLP---------FLLVASP-PNKADAYEHTITV
 8 sp|P89946.2|POLS  99.4%  50.7%     KSARADT-LDD-FSYLWTNNQAMFWLQLASPVAAFLCLSYCCRNLACCMKI--------FLGISGLCVIATQAYEHSTTM
 9 sp|P08768.1|POLS  99.0%  47.7%     KPTRADD-TLQVLNYLWNNNQNFFWMQTLIPLAALIVCMRMLAALFCCGPA--------FLLVCGAW---AAAYEHTAVM
10 sp|Q4QXJ7.1|POLS  99.2%  49.9%     KPTRADD-TLQVLNYLWNNNQNFFWMQTLIPLAALIVCMRMLRCLFCCGPA--------FLLVCGALG--AAAYEHTAVM
11 sp|Q306W5.1|POLS  99.2%  48.9%     KPTRADD-TLQVLNYLWNNNQNFFWMQTLIPLAALIVCMRMLRCLLCCGPA--------FLLVCGALG--AAAYEHTAVM
12 sp|Q306W7.1|POLS  99.2%  49.5%     KPTRADD-TLQVLNYLWNNNQNFFWMQTLIPLAALIVCMRMLRCLLCCGPA--------FLLVCGALG--AAAYEHAAVM
13 sp|P27284.1|POLS  99.0%  48.1%     KPTRADD-TLQVLNYLWNNNQNFFWMQTLIPLAALIVCMRIVRCLFCCGPA--------FLLVCGAW---AAAYEHTAVM
14 sp|Q5Y388.1|POLS  99.8%  59.5%     PRAHAAS-FAESMAYLWDENQTLFWLELATPLAAIIILVCCLKNLLCCCKPLS------FLVLVSLGTPVVKSYEHTATI
15 sp|Q8QZ72.1|POLS  99.2%  58.1%     PKAHAAS-FAEGMAYLWDNNQSMFWMELTGPLALLILATCCARSLLSCCKG-S------FLVAMSIGSAVASAYEHTAII
16 sp|Q80S27.1|POLS 100.0%  57.4%     PRAKAAT-FAETAAYLWAENQTVFWMQFAIPVACFMIVTYCLRHLMLCCRTAS------FLVAVSLGMGATQAYEHSVTL
17 sp|P27285.1|POLS  98.8%  45.7%     RSANAET-FTETMSYFWSNSQPFFWVQLCIPLAAVIVLMR---CCSCCLP---------FLVVAGAYLAKVDAYEHATTV
18 sp|P03316.1|POLS  98.8%  45.7%     RSANAET-FTETMSYLWSNSQPFFWVQLCIPLAAFIVLMR---CCSCCLP---------FLVVAGAYLAKVDAYEHATTV
19 sp|P13890.1|POLS  99.6%  57.3%     PRANAAS-FAETMAYLWDENKTLFWMEXXXXXXALALLACCIKSLICCCKPFS------FLVLLSLG-ASAKAYEHTATI
20 sp|P08491.3|POLS  99.6%  58.3%     PRANAAS-FAETMAYLWDENKTLFWMEFAAPAAALALLACCIKSLICCCKPFS------FLVLLSLG-ASAKAYEHTATI
21 sp|Q9JGK8.1|POLS  99.8%  58.7%     PRAHAAS-FAESMAYLWDENQTLFWLELATPLAAIIILVCCLKNLLCCCQPLS------FLVLVSLGTPVVKSYEHTATI
22 sp|Q8JJX0.1|POLS  99.8%  33.6%     PGARADQPYLDIIAYLWTNSKVAFGLQFAAPVACVLIITYALRHCRLCCKSFLGVRGWSALLVILAYVQSCKSYEHTVVV
23 sp|P03315.1|POLS  99.6%  59.3%     PRAHAAS-VAETMAYLWDQNQALFWLEFAAPVACILIITYCLRNVLCCCKSLS------FLVLLSLG-ATARAYEHSTVM
24 sp|Q8QL52.1|POLS 100.0%  33.8%     PGARADQPYLDIIAYLWTNSKVAFGLQCAAPVACMLIVTYALRHCRLCCNSFLGVRGWSALLVILAYVQSCKAYEHTVVV
25 sp|P36329.1|POLS  99.0%  49.8%     RTARAET-TWESLDHLWNNNQQMFWIQLLIPLAALIVVTRLLRCVCCVVP---------FLVVAGAAG--AGAYEHATTM
26 sp|P36330.1|POLS  99.0%  48.8%     RSARAET-TWESLDHLWNNNQQMFWTQLLIPLAALIVVTRLLKCMCCVVP---------FLVVAGAAG--AGAYEHATTM
27 sp|P36331.1|POLS  99.0%  50.0%     RTARAET-TWESLDHLWNHNQQMFWSQLLIPLAALIVATRLLKCVCCVVP---------FLVVAGAVG--AGAYEHATTM
28 sp|P36332.1|POLS  99.0%  49.4%     RTARAET-TWESLDHLWNNNQQMFWIQLLIPLAALIVVTRLLKCVCCVVP---------FLVVAGAAG--AGAYEHATTM
29 sp|P05674.1|POLS  98.8%  49.2%     RTARAET-TWESLDHLWNNNQQMFWIQLLIPLAALIVVTRLLRCVCCVVP---------FLVMAGAA---APAYEHATTM
30 sp|P09592.2|POLS  98.8%  49.2%     RTARAET-TWESLDHLWNNNQQMFWIQLLIPLAALIVVTRLLRCVCCVVP---------FLVMAGAA---AGAYEHATTM
31 sp|P13897.1|POLS  98.8%  42.8%     RPTNAET-FGETLNHLWFNNQPFLWAQLCIPLAALVILFR---CFSCCMP---------FLLVAGVCLGKVDAFEHATTV
   consensus/100%                     ..spAtp.h.p.hsahW.ppp.hhh.p......hhhhh......h..sh..........hLhh.......s.uaEHshhh
   consensus/90%                      ..spAts.hhpshsaLWsppp.hFWhphhhPlushllhh.....h.ssh..........FLhhhuhh...stAYEHsssh
   consensus/80%                      .ts+Ats.hh-shsaLWspsQshFWhQhhhPlAAhllhhphl+th.CCs..........FLhlhuhh...stAYEHsssh
   consensus/70%                      psA+Aso.hhEshsYLWspsQshFWhQhhIPLAAlIllsphL+sl.CCs..........FLllhuhhh..ssAYEHsssh

                              cov     pid  81         .         1         .         .         .         .         :         . 160
 1 sp|Q8JUX5.3|POLS 100.0% 100.0%     PNTVGVP-YKTLVNRPGYSPMVLEMELLSVTLEPTLSLDYITCEYKTVIPSPYVKCCGTAEC----------KDKNLPDY
 2 sp|Q5WQY5.1|POLS 100.0%  96.8%     PNTVGVP-YKTLVNRPGYSPMVLEMELLSVTLEPTLSLDYITCEYKTVIPSPYVKCLRYSEC----------KDKSLPDY
 3 sp|Q5XXP3.1|POLS 100.0%  96.4%     PNTVGVP-YKTLVNRPGYSPMVLEMELQSVTLEPTLSLDYITCEYKTVIPSPYVKCCGTAEC----------KDKSLPDY
```

```
 4 sp|P22056.1|POLS 100.0%  87.3%   PNTVGVP-CKTLVSRPGYSPMVLEMELQSVTLEPALSLDYITCEYKTITPSPYVKCCGTAEC----------KAKNLPDY
 5 sp|O90371.1|POLS 100.0%  88.1%   PNTVGVP-YKTLVSRPGYSPMVLEMELQSVTLEPTLFLDYITCEYKTITPSPYVKCCGTAEC----------KAKNLPDY
 6 sp|O90369.1|POLS 100.0%  87.9%   PNTVGVP-YKTLVSRPGYSPMVLEMELQSVTLEPTLFLDYITCEYKTITPSPYVKCCGTAEC----------KAKNLPDY
 7 sp|Q86925.2|POLS  98.4%  44.6%   PNAPLNS-YKALVERPGYAPLNLEVMVMNTQIIPSVKREYITCRYHTVVPSPQIKCCGTVEC----------PKGEKADY
 8 sp|P89946.2|POLS  99.4%  50.7%   PNQVGIP-FKALIERPGYAGLPLSLVVIKSELVPSLVQDYITCNYKTVVPSPYIKCCGGAEC----------SHKNEADY
 9 sp|P08768.1|POLS  99.0%  47.7%   PNKVGIP-YKALVERPGYAPVHLQIQLVNTRIIPSTNLEYITCKYKTKVPSPVVKCCGATQC----------TSKPHPDY
10 sp|Q4QXJ7.1|POLS  99.2%  49.9%   PNKVGIP-YKALVERPGYAPVHLQIQLVNTRIIPSTNLEYITCKYKTKVPSPVVKCCGATQC----------TSKPHPDY
11 sp|Q306W5.1|POLS  99.2%  48.9%   PNKVGIP-YKALVERPGYAPVHLQIQLVTTKIIPSANLEYITCKYKTKVPSPVVKCCGATQC----------TSKQHPDY
12 sp|Q306W7.1|POLS  99.2%  49.5%   PNKVGIP-YKALVERPGYAPVHLQIQLVTTKIIPSANLEYITCKYKTKVPSPVVKCCGSTQC----------SAKSLPDY
13 sp|P27284.1|POLS  99.0%  48.1%   PNKVGIP-YKALVERPGYAPVHLQIQLVNTSIIPSTNLEYITCKYKTKVPSPVVKCCGATQC----------TSKPHPDY
14 sp|Q5Y388.1|POLS  99.8%  59.5%   PNVVGFP-YKAHIERNGFSPMTLQLEVLGTSLEPTLNLEYITCEYKTVVPSPYIKCCGTSEC----------RSMERPDY
15 sp|Q8QZ72.1|POLS  99.2%  58.1%   PNQVGFP-YKAHVAREGYSPLTLQMQVIETSLEPTLNLEYITCDYKTKVPSPYVKCCGTAEC----------RTQDKPEY
16 sp|Q80S27.1|POLS 100.0%  57.4%   PNAVGFP-YRAHVDRPGFSPLTLHMEVVSTSLEPTLALDYVTCEYKTVVPSPKVTCCGMSEC----------AHQQKADF
17 sp|P27285.1|POLS  98.8%  45.7%   PNVPQIP-YKALVERAGYAPLNLEITVMSSEVLPSTNQEYITCKFTTVVPSPKVKCCGSLEC----------QPAAHADY
18 sp|P03316.1|POLS  98.8%  45.7%   PNVPQIP-YKALVERAGYAPLNLEITVMSSEVLPSTNQEYITCKFTTVVPSPKIKCCGSLEC----------QPAAHADY
19 sp|P13890.1|POLS  99.6%  57.3%   PNVVGFP-YKAHIERNXFSPMTLQLEVVXXSLEPTLNLEYITCEYKTVVPSPFIKCCGTSEC----------SSKEQPDY
20 sp|P08491.3|POLS  99.6%  58.3%   PNVVGFP-YKAHIERNGFSPMTLQLEVVETSWEPTLNLEYITCEYKTVVPSPFIKCCGTSEC----------SSKEQPDY
21 sp|Q9JGK8.1|POLS  99.8%  58.7%   PNVVGFP-YKAHIERNGFSPMTLQLEVLGTSLEPTLNLEYITCEYKTVVPPPYIKCCGASEC----------RSMERPDY
22 sp|Q8JJX0.1|POLS  99.8%  33.6%   PMDPRAPSYEAVINRNGYDPLKLTISVNFTVISPTTALEYWTCAGVPIVEPPHVGCCTSVSCPSDLSTLHAFTGKAVSDV
23 sp|P03315.1|POLS  99.6%  59.3%   PNVVGFP-YKAHIERPGYSPLTLQMQVVETSLEPTLNLEYITCEYKTVVPSPYVKCCGASEC----------STKEKPDY
24 sp|Q8QL52.1|POLS 100.0%  33.8%   PMDPRAPSYEAVINRNGYDPLKLTIAVNFTVISPTTALEYWTCAGVPVVEPPHVGCCTSVSCPSDLSTLHAFTGKAVSDV
25 sp|P36329.1|POLS  99.0%  49.8%   PSQAGIP-YNTIVNRAGYAPLPISITPTKIKLIPTVNLEYVTCHYKTGMDSPAIKCCGSQEC----------TPTYRPDE
26 sp|P36330.1|POLS  99.0%  48.8%   PNQAGIS-YNTIVNRAGYAPLPISITPTKIKLIPTVNLEYVTCHYKTGMDSPTIKCCGSQEC----------TPTYRPDE
27 sp|P36331.1|POLS  99.0%  50.0%   PNQVGIP-YNTIVNRAGYAPLPISIVPTKVKLIPTVNLEYITCHYKTGMDSPAIKCCGTQEC----------SPTYRPDE
28 sp|P36332.1|POLS  99.0%  49.4%   PSQAGIS-YNTIVNRAGYAPLPISITPTKIKLIPTVNLEYVTCHYKTGMDSPAIKCCGSQEC----------TPTNRPDE
29 sp|P05674.1|POLS  98.8%  49.2%   PSQAGIS-YNTIVNRAGYAPLPISITPTKIKLIPTVNLEYVTCHYKTGMDSPAIKCCGSQEC----------TPTYRPDE
30 sp|P09592.2|POLS  98.8%  49.2%   PSQAGIS-YNTIVNRAGYAPLPISITPTKIKLIPTVNLEYVTCHYKTGMDSPAIKCCGSQEC----------TPTYRPDE
31 sp|P13897.1|POLS  98.8%  42.8%   PNVPGIP-YKALVERAGYAPLNLEITVVSSELTPSTNKEYVTCRFHTVIPSPQVKCCGSLEC----------KASSKADY
   consensus/100%                   P..s..s.hpshltR..assh.lph......h.Psh..-YhTCthhshh.sP.ltCht..pC...............s-.
   consensus/90%                    Ps.sths.YpshlpRsGauPh.lpht..t.pl.Pohsh-YlTCcapThhsSPhlKCCGs.pC..........pst.hsD.
   consensus/80%                    PN.sGhP.YcshlpRsGYuPh.Lphplhpspl.PohsL-YlTCcYKThhsSPhlKCCGstpC..........psp.hsDh
   consensus/70%                    PNtsGhP.YKslVpRsGYuPhsLplplhpspl.PohsLEYITCcYKThlPSPhlKCCGosEC..........psptpPDY

                          cov    pid 161       .         .         .         2         .         .         .         . 240
 1 sp|Q8JUX5.3|POLS 100.0% 100.0%   SCKVFTGVYPFMWGGAYCFCDAENTQLSEAHVEKSESCKTE--FASAYRAHTASASAKLRVLYQGNNITVT-AYANGDHA
 2 sp|Q5WQY5.1|POLS 100.0%  96.8%   SCKVFTGVYPFMWGGAYCFCDTENTQLSEAHVEKSESCKTE--FASAYRAHTASASGKLRVLYQGNNVTVS-AYANGDHA
 3 sp|Q5XXP3.1|POLS 100.0%  96.4%   SCKVFTGVYPFMWGGAYCFCDAENTQLSEAHVEKSESCKTE--FASAYRAHTASASAKLRVLYQGNNITVA-AYANGDHA
 4 sp|P22056.1|POLS 100.0%  87.3%   NCKVFTGVYPFMWGGAYCFCDAENTQLSEAHVEKSESCKTE--FASAYRAHTASVSAKLRVFYQGNNITVS-AYANGDHA
 5 sp|O90371.1|POLS 100.0%  88.1%   NCKVFTGVYPFMWGGAYCFCDAENTQLSEAHVEKSESCKTE--FASAYRAHTASVSAKLRVFYQGNNITVS-AYANGDHA
 6 sp|O90369.1|POLS 100.0%  87.9%   NCKVFTGVYPFMWGGAYCFCDAENTQLSEAHVEKSESCKTE--FASAYRAHTASVSAKLRVFYQGNNITVS-AYANGDHA
 7 sp|Q86925.2|POLS  98.4%  44.6%   TCKVFTGVYPFLWGGAQCFCDSENSQLSDKYVELSTDCATD--HAEAVRVHTASVKSQLRITYGNSTAQVD-VFVNGVTP
 8 sp|P89946.2|POLS  99.4%  50.7%   KCSVFTGVYPFMWGGAYCFCDTENSQMSEVYVTRGESCEAD--HAIAYQVHTASLKAQVMISIGELNQTVD-VFVNGDSP
 9 sp|P08768.1|POLS  99.0%  47.7%   QCQVFTGVYPFMWGGAYCFCDTENTQMSEAYVERSEECSID--HAKAYKVHTGTVQAMVNITYGSVTWRSADVYVNGETP
10 sp|Q4QXJ7.1|POLS  99.2%  49.9%   QCQVFTGVYPFMWGGAYCFCDTENTQMSEAYVERSEECSID--HAKAYKVHTGTVQAMVNITYGSVSWRSADVYVNGETP
11 sp|Q306W5.1|POLS  99.2%  48.9%   QCQVFAGVYPFMWGGAYCFCDTENTQMSEAYIERAEECSVD--QAKAYKVHTGTVQAVVNITYGSVSWRSADVYVNGETP
12 sp|Q306W7.1|POLS  99.2%  49.5%   QCQVFTGVYPFMWGGAYCFCDTENTQMSEVYIERAEECSVD--QAKAYKVHTGTVQAVVNITYGSVSWRSADVYVNGETP
13 sp|P27284.1|POLS  99.0%  48.1%   QCQVFTGVYPFMWGGAYCFCDTENTQMSEAYVERSEECSID--HAKAYKVHTGTVQAMVNITYGSVSWRSADVYVNGETP
```

```
 14 sp|Q5Y388.1|POLS  99.8%  59.5%    QCQVYTGVYPFMWGGAYCFCDTENTQLSEAYVDRSDVCKHD--HAAAYKAHTAAMKATIRISYGNLNQTTT-AFVNGEHT
 15 sp|Q8QZ72.1|POLS  99.2%  58.1%    KCAVFTGVYPFMWGGAYCFCDSENTQMSEAYVERADVCKHD--HAAAYRAHTASLRAKIKVTYGTVNQTVE-AYVNGDHA
 16 sp|Q80S27.1|POLS 100.0%  57.4%    QCKVYTGVYPFLWGGAYCFCDSENTQLSEAYVERSEVCKHD--HAAAYRAHTAALKAKISVTYGSTNGTAE-AFVNGEST
 17 sp|P27285.1|POLS  98.8%  45.7%    TCKVFGGVYPFMWGGAQCFCDSENSQMSEAYVELSADCATD--HAQAIKVHTAAMKVGLRIVYGNTTSFLD-VYVNGVTP
 18 sp|P03316.1|POLS  98.8%  45.7%    TCKVFGGVYPFMWGGAQCFCDSENSQMSEAYVELSADCASD--HAQAIKVHTAAMKVGLRIVYGNTTSFLD-VYVNGVTP
 19 sp|P13890.1|POLS  99.6%  57.3%    QCKVYTGVYPFMWGGAYCFCDSENTQLSEAYVDRSDVCKHD--HALAYKAHTASLKATIRISYGTINQTTE-AFVNGEHA
 20 sp|P08491.3|POLS  99.6%  58.3%    QCKVYTGVYPFMWGGAYCFCDSENTQLSEAYVDRSDVCKHD--HASAYKAHTASLKATIRISYGTINQTTE-AFVNGEHA
 21 sp|Q9JGK8.1|POLS  99.8%  58.7%    QCQVYTGVYPFMWGGAYCFCDTENTQLSEAYVDRSDVCKHD--HAAAYKAHTAAMKATIRISYGNLNQTTT-AFVNGEHT
 22 sp|Q8JJX0.1|POLS  99.8%  33.6%    HCDVHTNVYPLLWGAAHCFCSTENTQVSAVAATVSEFCAQDSERAEAFSVHSSSVTAEVLVTLGEVVTAVH-VYVDGVTS
 23 sp|P03315.1|POLS  99.6%  59.3%    QCKVYTGVYPFMWGGAYCFCDSENTQLSEAYVDRSDVCRHD--HASAYKAHTASLKAKVRVMYGNVNQTVD-VYVNGDHA
 24 sp|Q8QL52.1|POLS 100.0%  33.8%    HCDVHTNVYPLLWGAAHCFCSTENTQVSAVAATVSEFCAQDSERAEAFSVHSSSVTAEILVTLGEVVTAVH-VYVDGVTS
 25 sp|P36329.1|POLS  99.0%  49.8%    QCKVFTGVYPFMWGGAYCFCDTENTQVSKAYVMKSDDCLAD--HAEAYKAHTASVQAFLNITVGEHSIVTT-VYVNGETP
 26 sp|P36330.1|POLS  99.0%  48.8%    QCKVFAGVYPFMWGGAYCFCDTENTQISKAYVMKSEDCLAD--HAAAYKAHTASVQALLNITVGEHSTVTT-VYVNGETP
 27 sp|P36331.1|POLS  99.0%  50.0%    QCKVFSGVYPFMWGGAYCFCDTENTQISKAYVTKSEDCVTD--HAQAYKAHTASVQAFLNITVGGHSTTAV-VYVNGETP
 28 sp|P36332.1|POLS  99.0%  49.4%    QCKVFTGVYPFMWGGAYCFCDTENTQVSKAYVMKSDDCLAD--HAEAYKAHTASVQAFLNITVGEHSIVTT-VYVNGETP
 29 sp|P05674.1|POLS  98.8%  49.2%    QCKVFTGVYPFMWGGAYCFCDTENTQVSKAYVMKSDDCLAD--HAEAYKAHTASVQAFLNITVGEHSIVTT-VYVNGETP
 30 sp|P09592.2|POLS  98.8%  49.2%    QCKVFTGVYPFMWGGAYCFCDTENTQVSKAYVMKSDDCLAD--HAEAYKAHTASVQAFLNITVGEHSIVTT-VYVNGETP
 31 sp|P13897.1|POLS  98.8%  42.8%    TCRVFGGVYPFMWGGAQCFCDSENTQLSEAYVEFAPDCTID--HAVALKVHTAALKVGLRIVYGNTTAHLD-TFVNGVTP
    consensus/100%                    pCtVassVYPhhWGuA.CFCssENoQhSthhh.hu..C..-...A.AhpsHoushps.l.l.htt.s.....sassG.ps
    consensus/90%                     pCpVasGVYPFhWGGA.CFCDsENoQhScsal.hup.C.h-..hAtAh+sHTushpu.lpl.htt.s.hst.sasNG.ps
    consensus/80%                     pCpVaTGVYPFMWGGAYCFCDoENTQhScAaVp+S-.CttD..aAtAY+sHTuohpAhlplhhGt.s.hss.saVNG-ps
    consensus/70%                     pCcVFTGVYPFMWGGAYCFCDoENTQlSEAYV-+S-sCttD..+ApAY+sHTAolpAhlplsYGshshsss.sYVNG-ps

                          cov    pid 241               :         .         .         .         .         3         .         . 320
  1 sp|Q8JUX5.3|POLS 100.0% 100.0%    VTVKDAKFIVGPMSSAWTPFDNKIVVYKGDVYNMDYPPFGAGRPGQFGDIQSRTP---ESKDVYANTQLVLQRPAAGTVH
  2 sp|Q5WQY5.1|POLS 100.0%  96.8%    VTVKDAKFIVGPMSSAWTPFDNKIVVYKGDVYNMDYPPFGAGRPGQFGDIQSRTP---ESEDVYANTQLVLQRPSAGTVH
  3 sp|Q5XXP3.1|POLS 100.0%  96.4%    VTVKDAKFVVGPMSSAWTPFDNKIVVYKGDVYNMDYPPFGAGRPGQFGDIQSRTP---ESKDVYANTQLVLQRPAAGTVH
  4 sp|P22056.1|POLS 100.0%  87.3%    VTVEDAKFVIGPLSSAWSPFDNKIVVYKGEVYNMDYPPFGAGRPGQFGDIQSRTP---DSKDVYANTQLILQRPAAGAIH
  5 sp|O90371.1|POLS 100.0%  88.1%    VTVKDAKFVIGPLSSAWSPFDNKIVVYKGEVYNMDYPPFGAGRPGQFGDIQSRTP---DSKDVYANTQLILQRPAAGAIH
  6 sp|O90369.1|POLS 100.0%  87.9%    VTVKDAKFVIGPLSSAWSPFDNKIVVYKGEVYNMDYPPFGAGRPGQFGDIQSRTP---DSKDVYANTQLILQRPAAGAIH
  7 sp|Q86925.2|POLS  98.4%  44.6%    ARSKDMKLIAGPLSTTFSPFDNKVIIYHGKVYNYDFPEFGAGTPGAFGDVQASST---TGSDLLANTAIHLQRPEARNIH
  8 sp|P89946.2|POLS  99.4%  50.7%    ARIQQSKFILGPISSAWSPFDHKVIVYRDEVYNEDYAPYGSGQAGRFGDIQSRTV---NSTDVYANTNLKLKRPASGNVH
  9 sp|P08768.1|POLS  99.0%  47.7%    AKIGDAKLIIGPLSSAWSPFDNKVVVYGHEVYNYDFPEYGTGKAGSFGDLQSRTS---TSNDLYANTNLKLQRPQAGIVH
 10 sp|Q4QXJ7.1|POLS  99.2%  49.9%    AKIGDAKLIIGPLSSAWSPFDNKVVVYGHEVYNYDFPEYGTGKAGSFGDLQSRTS---TSNDLYANTNLKLQRPQAGIVH
 11 sp|Q306W5.1|POLS  99.2%  48.9%    AKIGDAKLTIGPLSSAWTPFDSKVVVYGHEVYNYDFPEYGTGKAGSFGDLQSRTL---TSNDLYANTNLKLQRPQPGVVH
 12 sp|Q306W7.1|POLS  99.2%  49.5%    AKIGDAKLTIGPLSSAWSPFDSKVVVYGHEVYNYDFPEYGTGKAGSFGDLQSRTL---TSNDLYANTNLKLQRPQPGVVH
 13 sp|P27284.1|POLS  99.0%  48.1%    AKIGDAKLIIGPLSSAWSPFDNKVVVYGHEVYNYDFPEYGTGKAGSFGDLQSRTS---TSNDLYANTNLKLQRPQAGIVH
 14 sp|Q5Y388.1|POLS  99.8%  59.5%    VTVGGSRFTFGPISTAWTPFDNKIVVYKNDVYNQDFPPYGSGQPGRFGDIQSRTV---ESKDLYANTALKLSRPSSGTVH
 15 sp|Q8QZ72.1|POLS  99.2%  58.1%    VTIAGTKFIFGPVSTPWTPFDTKILVYKGELYNQDFPRYGAGQPGRFGDIQSRTL---DSRDLYANTGLKLARPAAGNIH
 16 sp|Q80S27.1|POLS 100.0%  57.4%    ARIGDLKMILGPISTAWSPFDPKIVVYKDEVYNQDYPPYGSGQPGRFGDLQSRTT---ESNDVYANTALKLARPSAGTVH
 17 sp|P27285.1|POLS  98.8%  45.7%    GTSKDLKVIAGPISASFTPFDHKVVIHRGLVYNYDFPEYGAMKPGVFGDIQATSL---TSKDLIASTDIRLLKPSAKNVH
 18 sp|P03316.1|POLS  98.8%  45.7%    GTSKDLKVIAGPISASFTPFDHKVVIHRGLVYNYDFPEYGAMKPGAFGDIQATSL---TSKDLIASTDIRLLKPSAKNVH
 19 sp|P13890.1|POLS  99.6%  57.3%    VNVGGSKFIFGPISTAWSPFDNKIVVYKDDVYNQDFPPYGSGQPGRFGDIQSRTV---ESKDLYANTALKLSRPSPGVVH
 20 sp|P08491.3|POLS  99.6%  58.3%    VNVGGSKFIFGPISTAWSPFDNKIVVYKDDVYNQDFPPYGSGQPGRFGDIQSRTV---ESKDLYANTALKLSRPSPGVVH
 21 sp|Q9JGK8.1|POLS  99.8%  58.7%    VTVGGSRFTFGPISTAWTPFDNKIVVYKNDVYNQDFPPYGSGQPGRFGDVQSRTV---ESKDLYANTALKLSRPSSGTVH
 22 sp|Q8JJX0.1|POLS  99.8%  33.6%    ARGTDLKIVAGPITTDYSPFDRKVVRIGEEVYNYDWPPYGAGRPGTFGDIQARSTNYVKPNDLYGDIGIEVLQPTNDHVH
 23 sp|P03315.1|POLS  99.6%  59.3%    VTIGGTQFIFGPLSSAWTPFDNKIVVYKDEVFNQDFPPYGSGQPGRFGDIQSRTV---ESNDLYANTALKLARPSPGMVH
```

```
24 sp|Q8QL52.1|POLS 100.0%  33.8%     ARGTDLKIVAGPITTDYSPFDRKVVRIGEEVYNYDWPPYGAGRPGTFGDIQARSTNYVKPNDLYGDIGIEVLQPTNDHVH
25 sp|P36329.1|POLS  99.0%  49.8%     VNFNGVKLTAGPLSTAWTPFDRKIVQYAGEIYNYDFPEYGAGQPGAFGDIQSRTV---SSSDLYANTNLVLQRPKAGAIH
26 sp|P36330.1|POLS  99.0%  48.8%     VNFNGVKLTAGPLSTAWTPFDRKIVQYAGEIYNYDFPEYGAGQPGAFGDIQLRTV---SSSDLYANTNLVLQRPKAGAIH
27 sp|P36331.1|POLS  99.0%  50.0%     VNFNGVKLTAGPLSTAWSPFDKKIVQYAGEIYNYDFPEYGAGHAGAFGDIQARTI---SSSDVYANTNLVLQRPKAGAIH
28 sp|P36332.1|POLS  99.0%  49.4%     VNFNGVKLTAGPLSTAWTPFDRKIVQYAGEIYNYDFPEYGAGQPGAFGDIQSRTV---SSSDLYANTNLVLQRPKAGAIH
29 sp|P05674.1|POLS  98.8%  49.2%     VNFNGVKITAGPLSTAWTPFDRKIVQYAGEIYNYDFPEYGAGQPGAFGDIQSRTV---SSSDLYANTNLVLQRPKAGAIH
30 sp|P09592.2|POLS  98.8%  49.2%     VNFNGVKLTAGPLSTAWTPFDRKIVQYAGEIYNYDFPEYGAGQPGAFGDIQSRTV---SSSDLYANTNLVLQRPKAGAIH
31 sp|P13897.1|POLS  98.8%  42.8%     GSSRDLKVIAGPISAAFSPFDHKVVIRKGLVYNYDFPEYGAMKPGAFGDIQASSL---DATDIVARTDIRLLKPSVKNIH
   consensus/100%                     sp.tt.phhhGPhossaoPFD.Kll.htt.laN.Das.aGshpsG.FGDlQ.po....pspDlhuphtl.l.pPtst.lH
   consensus/90%                      sp.ts.KhhhGPlSosaoPFDpKlV.attclYN.DaP.aGsGpsGtFGDlQupo....pupDlhAsTtlhL.+Ptst.lH
   consensus/80%                      sphtshKhhhGPlSoAWoPFDpKlVhYtt-lYN.DaP.aGuGpsGtFGDlQuRT....pSpDlYANTsLhLtRPtsGslH
   consensus/70%                      sphtssKhhhGPlSoAWoPFDpKlVlYts-VYNhDaP.YGuGpPGtFGDIQSRTs...pSpDlYANTsLhLpRPpuGslH

                      cov    pid 321               .           .           :           .           .           .           .         4 400
 1 sp|Q8JUX5.3|POLS 100.0% 100.0%     VPYSQAPSGFKYWLKERGASLQHTAPFGCQIATNPVRAMNCAVGNMPISIDIPDAAFTRVVDAPSLTDMSCEVPACTHSS
 2 sp|Q5WQY5.1|POLS 100.0%  96.8%     VPYSQAPSGFKYWLKERGASLQHTAPFGCQIATNPVRAMNCAVGNMPISIDIPDAAFTRVVDAPSLTDMSCEVPACTHSS
 3 sp|Q5XXP3.1|POLS 100.0%  96.4%     VPYSQAPSGFKYWLKERGASLQHTAPFGCQIATNPVRAVNCAVGNIPISIDIPDAAFTRVVDAPSVTDMSCEVPACTHSS
 4 sp|P22056.1|POLS 100.0%  87.3%     VPYSQAPSGFKYWLKEKGASLQHTAPFGCQIATNPVRAVNCAVGNIPVSIDIPDAAFTRVTDAPSITDMSCEVASCTHSS
 5 sp|O90371.1|POLS 100.0%  88.1%     VPYSQAPSGFKYWLKEKGASLQHTAPFGCQIATNPVRAVNCAVGNIPVSIDIPDAAFTRVTDAPSVTDMSCEVASCTHSS
 6 sp|O90369.1|POLS 100.0%  87.9%     VPYSQAPSGFKYWLKEKGASLQHTAPFGCQIATNPVRAVNCAVGNIPVSIDIPDAAFTRVTDAPSVTDMSCEVASCTHSS
 7 sp|Q86925.2|POLS  98.4%  44.6%     VPYTQAPSGFEFWKNNSGQPLSDTAPFGCKVNVNPLRADKCAVGSLPISVDIPDAAFTRVS-EPLPSLLKCTVTSCTYST
 8 sp|P89946.2|POLS  99.4%  50.7%     VPYTQTPSGFSYWKKEKGVPLNRNAPFGCIIKVNPVRAENCVYGNIPISMDIADAHFTRIDESPSVSLKACEVQSCTYSS
 9 sp|P08768.1|POLS  99.0%  47.7%     TPFTQAPSGFERWKRDKGAPLNDVAPFGCSIALEPLRPENCAVGSIPISIDIPDAAFTRISETPTVSDLECKITECTYAS
10 sp|Q4QXJ7.1|POLS  99.2%  49.9%     TPFTQAPSGFERWKRDKGAPLNDVAPFGCSIALEPLRAENCAVGSIPISIDIPDAAFTRISETPTVSDLECKITECTYAS
11 sp|Q306W5.1|POLS  99.2%  48.9%     TPYTQAPSGFERWKKDRGAPLNDIAPFGCTIALDPLRAENCAVGNIPLSIDIPDAAFTRISETPTVSDLECKITECTYAS
12 sp|Q306W7.1|POLS  99.2%  49.5%     TPYTQAPSGFERWKKDRGAPLNDIAPFGCTIALDPLRAENCAVGNIPLSIDIPDAAFTRIAETPTVSDLECKVTECTYAS
13 sp|P27284.1|POLS  99.0%  48.1%     TPFTQAPSGFERWKRDKGAPLNDVAPFGCSIALEPLRAENCAVGSIPISIDIPDAAFTRISETPTVSDLECKITECTYAS
14 sp|Q5Y388.1|POLS  99.8%  59.5%     VPYTQTPSGFKYWIKERGTSLNDKAPFGCVIKTNPVRAENCAVGNIPVSMDIPDTAFTRVIDAPAVTNLECQVAVCTHSS
15 sp|Q8QZ72.1|POLS  99.2%  58.1%     VPYTQTPSGFKTWQKDRDSPLNAKAPFGCIIQTNPVRAMNCAVGNIPVSMDIADSAFTRLTDAPVISELTCTVSTCTHSS
16 sp|Q80S27.1|POLS 100.0%  57.4%     VPYTQTPSGFKYWLKEKGDALNHKAPFGCIIKTNPVRAENCAVGNIPVSLDIPDAAFTRIVDAPSLTGLKCEVATCTHSS
17 sp|P27285.1|POLS  98.8%  45.7%     VPYTQAASGFEMWKNNSGRPLQETAPFGCKIAVNPLRAVDCSYGNIPISIDIPNAAFIRTSDAPLVSTVKCDVSECTYSA
18 sp|P03316.1|POLS  98.8%  45.7%     VPYTQASSGFEMWKNNSGRPLQETAPFGCKIAVNPLRAVDCSYGNIPISIDIPNAAFIRTSDAPLVSTVKCEVSECTYSA
19 sp|P13890.1|POLS  99.6%  57.3%     VPYTQTPSGFKYWLKEKGSSLNTKAPFGCKIKTNPVRAMDCAVGSIPVSMDIPDSAFTRVVDAPAVTDLSCQVAVCTHSS
20 sp|P08491.3|POLS  99.6%  58.3%     VPYTPTPSGFKYWLKEKGSSLNTKAPFGCKIKTNPVRAMDCAVGSIPVSMDIPDSAFTRVVDAPAVTDLSCQVVVCTHSS
21 sp|Q9JGK8.1|POLS  99.8%  58.7%     VPYTQTPSGFKYWIKERGTSLNDKAPFGCVIKTNPVRAENCAVGNIPVSMDIPDSAFTRVIDAPAVTNLECQVAVCTHSS
22 sp|Q8JJX0.1|POLS  99.8%  33.6%     VAYTYTTSGLLRWLQDAPKPLSVTAPHGCKISANPLLALDCGVGAVPMSINIPDAKFTRKLKDPKPSALKCVVDSCEYGV
23 sp|P03315.1|POLS  99.6%  59.3%     VPYTQTPSGFKYWLKEKGTALNTKAPFGCQIKTNPVRAMNCAVGNIPVSMNLPDSAFTRIVEAPTIIDLTCTVATCTHSS
24 sp|Q8QL52.1|POLS 100.0%  33.8%     VAYTYTTSGLLRWLQDAPKPLSVTAPHGCKISANPLLALDCGVGAVPMSINIPDAKFTRKLKDPKPSALKCVVDSCEYGV
25 sp|P36329.1|POLS  99.0%  49.8%     VPYTQAPSGFEQWKKDKAPSLKFTAPFGCEIYTNPIRAENCAVGSIPLAFDIPDALFTRVSETPTLSAAECTLNECVYSS
26 sp|P36330.1|POLS  99.0%  48.8%     VPYTQAPSGFEQWKKDKAPSLKFTAPFGCEIYTNPIRAENCAVGSIPLAFDIPDALFTRVSETPTLSAAECTLNECVYSS
27 sp|P36331.1|POLS  99.0%  50.0%     VPYTQAPSGYEQWKKDKPPSLKFTAPFGCEIYTNPIRAENCAVGSIPLAFDIPDALFTRVSETPTLSTAECTLNECVYSS
28 sp|P36332.1|POLS  99.0%  49.4%     VPYTQAPSGFEQWKKDKAPSLKFTAPFGCEIYTNPIRAENCAVGSIPLAFDIPDALFTRVSETPTLSAAECTLNECVYSS
29 sp|P05674.1|POLS  98.8%  49.2%     VPYTQAPSGFEQWKKDKAPSLKFTAPFGCEIYTNPIRAENCAVGSIPLAFDIPDALFTRVSETPTLSAAECTLNECVYSS
30 sp|P09592.2|POLS  98.8%  49.2%     VPYTQAPSGFEQWKKDKAPSLKFTAPFGCEIYTNPIRAENCAVGSIPLAFDIPDALFTRVSETPTLSAAECTLNECVYSS
31 sp|P13897.1|POLS  98.8%  42.8%     VPYTQAVSGYEMWKNNSGRPLQETAPFGCKIEVEPLRASNCAYGHIPISIDIPDAAFVRSSESPTILEVSCTVADCIYSA
   consensus/100%                     ssao.ssSGh..W.ppts.sLp..APaGC.l.hpPlhs.pCshGthPhuhslssshFhR...tP....htC.l..C.aus
   consensus/90%                      sPYoQssSGac.Whppps.sLp.hAPFGC.I.hpPlRA.sCuhGslPluhDIPDuhFTRh.-sP.lothpCpls.Csauu
```

```
      consensus/80%                        VPYTQsPSGFchWh+-+ussLp.pAPFGCpItsNPlRA.sCAVGsIPlShDIPDAtFTRls-sPsloshpCplstCsauS
      consensus/70%                        VPYTQsPSGFchWhK-+GssLppsAPFGCpItsNPlRA.NCAVGsIPlShDIPDAAFTRls-sPsloshpCplspCTaSS

                                 cov    pid 401      .         .         .         .         :         .         .         . 480
   1 sp|Q8JUX5.3|POLS 100.0% 100.0%        DFGGVAIIKYAVSKKGKCAVHSMTNAVTIREAEIEVEG-NSQLQISFSTALASAEFRVQVCSTQVHCAAECHPPKDHIVN
   2 sp|Q5WQY5.1|POLS 100.0%  96.8%        DFGGAAIIKYAASKKGKCAVHSMTNAVTIREAEIEVEG-NSQLQISFSTALASAEFRVQVCSTQVHCAAECHPPKDHIVN
   3 sp|Q5XXP3.1|POLS 100.0%  96.4%        DFGGVAIIKYTASKKGKCAVHSMTNAVTIREADVEVEG-NSQLQISFSTALASAEFRVQVCSTQVHCAAACHPPKDHIVN
   4 sp|P22056.1|POLS 100.0%  87.3%        DFGGAAVIKYTASKKGKCAVHSVTNAVTIREPNVDVKG-TAQLQIAFSTALASAEFKVQICSTLVHCSATCHPPKDHIVN
   5 sp|O90371.1|POLS 100.0%  88.1%        DFGGAAVIKYTASKKGKCAVHSLTNAVTIREPNVDVEG-TAQLQIAFSTALASAEFKVQICSTQVHCSATCHPPKDHIVN
   6 sp|O90369.1|POLS 100.0%  87.9%        DFGGAAVVKYTASKKGKCAVHSLTNAVTIREPNVDVEG-TAQLQIAFSTALASAEFKVQICSTQVHCSATCHPPKDHIVN
   7 sp|Q86925.2|POLS  98.4%  44.6%        DYGGVLVLTYESDRAGQCAVHSHSSTAVLRDPSVYVEQ-KGETTLKFSTRSLQADFEVSMCGTRTTCHAQCQPPTEHVMN
   8 sp|P89946.2|POLS  99.4%  50.7%        DFGGVASISYTSNKVGKCAIHSHSNSATMKDSVQDVQE-SGALSLFFATSSVEPNFVVQVCNARITCHGKCEPPKDHIVP
   9 sp|P08768.1|POLS  99.0%  47.7%        DFGGIATLPTNPVKQETVQFIVHQVLQLLKRMTSPLLR-AGSFTFHFSTANIHPAFKLQVCTSGITCKGDCKPPKDHIVD
  10 sp|Q4QXJ7.1|POLS  99.2%  49.9%        DFGGIATVAYKSSKAGNCPIHSPSGVAVIKENDVTLAE-SGSFTFHFSTANIHPAFKLQVCTSAVTCKGDCKPPKDHIVD
  11 sp|Q306W5.1|POLS  99.2%  48.9%        DFGGIATVAYKASKAGNCPIHSPSGIAVIKENDVTLAD-SGSFTFHFSTASIHPAFKMQICTSVVTCKGDCKPPKDHIVD
  12 sp|Q306W7.1|POLS  99.2%  49.5%        DFGGIATISYKASKAGNCPIHSPSGIAVIKENDVTLAD-SGAFTFHFSTASIHPAFKMQVCTSVVTCKGDCKPPKDHIVD
  13 sp|P27284.1|POLS  99.0%  48.1%        DFGGIATLPTNPVKQETVQFILHQVLQLLKRMTSPLLR-AGSFTFHFSTANIHPAFKLQVCTSGVTCKGDCKPPKDHIVD
  14 sp|Q5Y388.1|POLS  99.8%  59.5%        DFGGIATLTFKTDKPGKCAVHSHSNVATIQEAAVDIKT-DGKITLHFSTASASPAFKVSVCSAKTTCMAACEPPKDHIVP
  15 sp|Q8QZ72.1|POLS  99.2%  58.1%        DFGGIAVLSYKVEKSGRCDIHSHSNVAVLQEVSIETE---GRSVIHFSTASASPSFVVSVCSSRATCTAKCEPPKDHVVT
  16 sp|Q80S27.1|POLS 100.0%  57.4%        DFGGTLVVEYKTDKVGTCAVHSESNTAVMQETSLSVTM-DGRGTLHFSTASASPSFVLKVCSSKTTCTAKCVPPKDHVVP
  17 sp|P27285.1|POLS  98.8%  45.7%        DFGGMATLQYVSDREGQCPVHSHSSTATLQESTVHVLE-KGAVTVHFSTASPQANFIVSLCGKKTTCNAECKPPADHIVS
  18 sp|P03316.1|POLS  98.8%  45.7%        DFGGMATLQYVSDREGQCPVHSHSSTATLQESTVHVLE-KGAVTVHFSTASPQANFIVSLCGKKTTCNAECKPPADHIVS
  19 sp|P13890.1|POLS  99.6%  57.3%        DFGXVATLSYKTDKPGKCAVHSHSNVATLQEATVDVKE-DGKVTVHFSXXSASPAFKVSVCDAKTTCTAACEPPKDHIVP
  20 sp|P08491.3|POLS  99.6%  58.3%        DFGGVATLSYKTDKPGKCAVHSHSNVATLQEATVDVKE-DGKVTVHFSTASASPAFKVSVCDAKTTCTAACEPPKDHIVP
  21 sp|Q9JGK8.1|POLS  99.8%  58.7%        DFGGIATLTFKTDKPGKCAVHSHSNVATIQEAAVDIKT-DGKITLHFSTASASPAFMVSVCSAKTTCMAACEPPKDHIVP
  22 sp|Q8JJX0.1|POLS  99.8%  33.6%        DYGGAATITYEGHEAGKCGIHSLTPGVPLRTSVVEVVAGANTVKTTFSSPTPEVALEVEICSAIVKCAGECTPPKEHVVA
  23 sp|P03315.1|POLS  99.6%  59.3%        DFGGVLTLTYKTNKNGDCSVHSHSNVATLQEATAKVKT-AGKVTLHFSTASASPSFVVSLCSARATCSASCEPPKDHIVP
  24 sp|Q8QL52.1|POLS 100.0%  33.8%        DYGGAATITYEGHEAGKCGIHSLTPGVPLRTSVVEVVAGANTVKTTFSSPTPEVTLEVEICSAIVKCASECTPPKEHVVA
  25 sp|P36329.1|POLS  99.0%  49.8%        DFGGIATVKYSASKSGKCAVHVPSGTATLKEAAIELAE-QGSATIHFSTANIHPEFRLQICTSYVTCKGDCHPPKDHIVT
  26 sp|P36330.1|POLS  99.0%  48.8%        DFGGIATVKYSASKSGKCAVHVPSGTATLKEASVELAE-QGSVTIHFSTANIHPEFRLQICTSFVTCKGDCHPPKDHIVT
  27 sp|P36331.1|POLS  99.0%  50.0%        DFGGIATVKYSASKSGKCAVHVPSGTATLKEAAVELAE-QGSATIHFSTASIHPEFRLQICTSYVTCKGDCHPPKDHIVT
  28 sp|P36332.1|POLS  99.0%  49.4%        DFGGIATVKYSASKSGKCAVHVPSGTATLKEAAVELTE-QGSATIHFSTANIHPEFRLQICTSYVTCKGDCHPPKDHIVT
  29 sp|P05674.1|POLS  98.8%  49.2%        DFGGIATVKYSASKSGKCAVHVPSGTATLKEAAVELTE-QGSATIHFSTANIHPEFRLQICTSYVTCKGDCHPPKDHIVT
  30 sp|P09592.2|POLS  98.8%  49.2%        DFGGIATVKYSASKSGKCAVHVPSGTATLKEAAVELTE-QGSATIHFSTANIHPEFRLQICTSYVTCKGDCHPPKDHIVT
  31 sp|P13897.1|POLS  98.8%  42.8%        DFGGSLTLQYKADREGHCPVHSHSTTAVLKEATTHVTA-VGSITLHFSTSSPQANFIVSLCGKKTTCNAECKPPADHIIG
      consensus/100%                       DaG..h.l.h.s.c.tps.hh..ps...hpp....h....st..h.Fu....psth.hphCst.hpC.utC.PPt-Hlhs
      consensus/90%                        DFGGhhsltatss+.GpCslHs.oshsslpcsshpl.t.tuthphtFSTs..pstFhlplCss.spCtutCpPPpDHlVs
      consensus/80%                        DFGGhAslpYpssKtGpCslHs.osssslpEsslpltt.tuphphpFSTAshpstFhlplCsshspCtupCcPPKDHIVs
      consensus/70%                        DFGGhAolpYpssKtGcCuVHS.osssssl+Esslcltt.pGphol+FSTAshpstFclplCoshsTCpupCcPPKDHIVs

                                 cov    pid 481      .         5         .         .         .       ] 536
   1 sp|Q8JUX5.3|POLS 100.0% 100.0%        YPASHTTLGVQDISATAMSWVQKITGGVGLVVAVAALILIVVLCVSFSRH------
   2 sp|Q5WQY5.1|POLS 100.0%  96.8%        YPASHTTLGVQDISATAMSWVQKITGGVGLVVAVAALILIVVLCVSFSRH------
   3 sp|Q5XXP3.1|POLS 100.0%  96.4%        YPASHTTLGVQDISTTAMSWVQKITGGVGLIVAVAALILIVVLCVSFSRH------
   4 sp|P22056.1|POLS 100.0%  87.3%        YPSPHTTLGVQDISTTAMSWVQKITGGVGLVVAIAALILIIVLCVSFSRH------
   5 sp|O90371.1|POLS 100.0%  88.1%        YPSPHTTLGVQDISTTAMSWVQKITGGVGLVVAIAALILIIVLCVSFSRH------
   6 sp|O90369.1|POLS 100.0%  87.9%        YPSPHTTLGVQDISTTAMSWVQKITGGVGLVVAIAALILIIVLCVSFSRH------
```

```
 7 sp|Q86925.2|POLS  98.4%  44.6%   RPQKSTPDFSSAISKTSWNWITALMGGISSIAAIAAIVLVIALVFTAQHR------
 8 sp|P89946.2|POLS  99.4%  50.7%   YAAKHNDAEFPSISTTAWQWLAHTTSGPLTILVVAIIVVVVVSIVVCARH------
 9 sp|P08768.1|POLS  99.0%  47.7%   YPAQHTESFTSAISATAWSWLKVLVGGTSAFIVLGLIATAVVALVLFFHRH-----
10 sp|Q4QXJ7.1|POLS  99.2%  49.9%   YPAQHTESFTSAISATAWSWLKVLVGGTSAFIVLGLIATAVVALVLFFHRH-----
11 sp|Q306W5.1|POLS  99.2%  48.9%   YPAQHTETFTSAVSATAWSWLKVLVGSTSAFIVLGLIATAVVALVLFTHKH-----
12 sp|Q306W7.1|POLS  99.2%  49.5%   YPAQHTETYTSAVSATAWSWLKVLVGSTSAFIVLGLIATAVVALVLFTHRH-----
13 sp|P27284.1|POLS  99.0%  48.1%   YPAQHTESFTSAISATAWSWLKVLVGGTSAFIVLGLIATAVVALVLFFHRH-----
14 sp|Q5Y388.1|POLS  99.8%  59.5%   YGASHNNQVFPDMSGTAMTWVQRVAGGLGGLTLAAVAVLILVTCVTMRR-------
15 sp|Q8QZ72.1|POLS  99.2%  58.1%   YPANHNGVTLPDLSSTAMTWAQHLAGGVGLLIALAVLILVIVTCVTLRR-------
16 sp|Q80S27.1|POLS 100.0%  57.4%   FPANHNNVVFPDFSSTAVSWLTHTMGGATVVIAIGITIFLIVTCIAFSRH------
17 sp|P27285.1|POLS  98.8%  45.7%   TPHKNDQEFQAAISKTSWSWLFALFGGASSLLIIGLTIFACSMMLTSTRR------
18 sp|P03316.1|POLS  98.8%  45.7%   TPHKNDQEFQAAISKTSWSWLFALFGGASSLLIIGLMIFACSMMLTSTRR------
19 sp|P13890.1|POLS  99.6%  57.3%   YGASHNNQVFPDMSGTAMTWVQRMASGLGGLALIAVVVLVLVTCITMRR-------
20 sp|P08491.3|POLS  99.6%  58.3%   YGASHNNQVFPDMSGTAMTWVQRLASGLGGLALIAVVVLVLVTCITMRR-------
21 sp|Q9JGK8.1|POLS  99.8%  58.7%   YGASHNNQVFPDMSGTAMTWVQRVAGGLGGLTLAAVAALILVTCVTMRR-------
22 sp|Q8JJX0.1|POLS  99.8%  33.6%   TRPRHGSDPGGYISGPAMRWAGGIVG-TLVVLFLILAVIYCVVKKCRSKRIRIVKS
23 sp|P03315.1|POLS  99.6%  59.3%   YAASHSNVVFPDMSGTALSWVQKISGGLGAFAIGAILVLVVVTCIGLRR-------
24 sp|Q8QL52.1|POLS 100.0%  33.8%   ARPRHGSDTGGYISGPAMRWAGRIVGNPSGPVSSSLAVTYCVVKKCRSKRIRIVKS
25 sp|P36329.1|POLS  99.0%  49.8%   HPQYHAQTFTAAVSKTAWTWLTSLLGGSAVIIIIGLVLATIVAMYVLTNQKHN---
26 sp|P36330.1|POLS  99.0%  48.8%   HPQYHAQTFTAAVSKTAWTWLTSLLGGSAVIIIIGLVLATLVAMYVLTNQKHN---
27 sp|P36331.1|POLS  99.0%  50.0%   HPQYHAQSFTAAVSKTAWTWLTSLLGGSAIIIIIGLVLATIVAMYVLTNQKHN---
28 sp|P36332.1|POLS  99.0%  49.4%   HPQYHAQTFTAAVSKTAWTWLTSLLGGSAVIIIIGLVLATIVAMYVLTNQKHN---
29 sp|P05674.1|POLS  98.8%  49.2%   HPQYHAQTFTAAVSKTAWTWLTSLLGGSAVIIIIGLVLATIVAMYVLTNQKHN---
30 sp|P09592.2|POLS  98.8%  49.2%   HPQYHAQTFTAAVSKTAWTWLTSLLGGSAVIIIIGLVLATIVAMYVLTNQKHN---
31 sp|P13897.1|POLS  98.8%  42.8%   EPHKVDQEFQAAVSKTSWNWLLALFGGASSLIVVGLIVLVCSSMLINTRR------
   consensus/100%                   .....s......hStsuhpWh..h.u.....h...hhhhhhs.hh...p.......
   consensus/90%                    hst.psp.h..shStTuhsWlt.lhGu.u.hhhhuhhhhhhshhh.htp.......
   consensus/80%                    ast.Hsp.hhsslStTAhoWlptlhGGsuhhlhluhhlhhlVhhhshp+p......
   consensus/70%                    aPtpHspthhsslStTAhoWlptlhGGsusllhlulllhhlVshlshp+p......
```

MView 1.63, Copyright © 1997-2018 Nigel P. Brown
