# Supplementary material for: Chikungunya Virus Diagnosis: A Review of Current Antigen Detection Methods
Source: Trop Med Infect Dis. 2023 Jul 17;8(7):365. doi: 10.3390/tropicalmed8070365 (PMC10383795; doi:10.3390/tropicalmed8070365)
Supplement: Supplementary file 1 [file tropicalmed-08-00365-s001.zip › MView-E2-Alignment.pdf]

```
Reference sequence (1): sp|Q8JUX5.3|POLS
Identities normalised by aligned length.
Colored by: identity

                                   cov    pid   1 [         .         .         .         .         :         .         .         . 80
  1 sp|Q8JUX5.3|POLS  100.0% 100.0%     PYLAHCPDCGEGHSCHSPVALERIRNEATDGTLKIQVSLQIGIG-TDDSHDWTKLRYMDNHIPADAG---RAGLFVRTSA
  2 sp|Q5WQY5.1|POLS  100.0%  95.3%     PYLAHCPDCGEGHSCHSPVALERIRNEATDGTLKIQVSLQIGIK-TDDSHDWTKLRYMDNHMPADAE---RAGLLVRTSA
  3 sp|P0DOK1.1|POLSF 100.0%  99.5%     PYLAHCPDCGEGHSCHSPVALERIRNEATDGTLKIQVSLQIGIG-TDDSHDWTKLRYMDNHIPADAG---RAGLFVRTSA
  4 sp|Q5XXP3.1|POLS  100.0%  92.8%     PYLAHCPDCGEGHSCHSPIALERIRNEATDGTLKIQVSLQIGIK-TDDSHDWTKLRYMDSHTPADAE---RAGLLVRTSA
  5 sp|P22056.1|POLS  100.0%  82.7%     PYLAHCPDCGEGHSCHSPIALERIRSEATDGTLKIQVSLQIGIK-TDDSHDWTKLRYMDSHTPVDAD---RSGLFVRTSA
  6 sp|O90371.1|POLS  100.0%  82.9%     PYLAHCPDCGEGHSCHSPIALERIRSEATDGTLKIQVSLQIGIK-TADSHDWTKLRYMDSHTPVDAD---RSGLFVRTSA
  7 sp|O90369.1|POLS  100.0%  82.9%     PYLAHCPDCGEGHSCHSPIALERIRSEATDGTLKIQVSLQIGIK-TDDSHDWTKLRYMDSHTPVDAD---RSGLFVRTSA
  8 sp|Q86925.2|POLS   99.3%  35.9%     PYLGLCHRCKTMEPCYSPIKIEKVWDDADDGVLRIQVSAQLGYN-RAGTAASARLRFMGGGVPPEIQEGAIADFKVFTSK
  9 sp|P89946.2|POLS   99.3%  40.9%     PYIGWCADCGLAGSCPSPVSIEHVWSDADDGVLKIQVSMQIGIA-KSNTINHAKIRYMGANGVQEAE---RSTLSVSTTA
 10 sp|P08768.1|POLS   98.8%  38.7%     PYIADCPNCGHSR-CDSPIAIEEVRGDAHAGVIRIQTSAMFGLK-RHG-VDLAYMSFMNGKTQKSIK---IDNLHVRTSA
 11 sp|Q4QXJ7.1|POLS   98.8%  39.7%     PYIADCPNCGHSR-CDSPIAIEEVRGDAHAGVIRIQTSAMFGLK-TDG-VDLAYMSFMNGKTQKSIK---IDNLHVRTSA
 12 sp|Q306W5.1|POLS   98.8%  37.7%     PYIADCSNCGHGR-CDSPIAIEDVRGNAHAGYIRIQTSAMFGLK-SDG-VDLAYMSFMNGKTLKAIK---IEHLYARTSA
 13 sp|Q306W7.1|POLS   98.8%  38.4%     PYIADCSNCGHGR-CDSPIAIEDIRGDAHAGYIRIQTSAMFGLK-SDG-VDLAYMSFMNGKTLKAIK---IEHLYARTSA
 14 sp|P27284.1|POLS   98.8%  39.7%     PYIADCPNCGHSR-CDSPIAIEEVRGDAHAGVIRIQTSAMFGLK-TDG-VDLAYMSFMNGKTQKSIK---IDNLHVRTSA
 15 sp|Q5Y388.1|POLS   99.8%  53.7%     PYLAYCADCGDGQFCYSPVAIEKIRDEASDGMIKIQVAAQIGIN-KGGTHEHNKIRYIAGHDMKEAN---RDSLQVHTSG
 16 sp|Q8QZ72.1|POLS   99.8%  55.4%     PYVAYCADCGMGHSCHSPAMIENIQADATDGTLKIQFASQIGLT-KTDTHDHTKIRYAEGHDIAEAA---RSTLKVHSSS
 17 sp|Q80S27.1|POLS   99.5%  51.0%     PYIAYCSDCGNGQFCYSPIAIERVRAEASDGMLKIQISAQIGLQ-VDGAHSWTKIRYMKGHDVEDTD---RNSLEVFTTG
 18 sp|P27285.1|POLS   99.3%  36.3%     PYLGTCSYCHHTEPCFSPIKIEQVWDEADDNTIRIQTSAQFGYD-KSGAASTNKYRYMSFEQDHTVKEGTMDDIKISTSG
 19 sp|P03316.1|POLS   99.3%  36.6%     PYLGTCSYCHHTVPCFSPVKIEQVWDEADDNTIRIQTSAQFGYD-QSGAASANKYRYMSLKQDHTVKEGTMDDIKISTSG
 20 sp|P0DOK0.1|POLSF  99.3%  36.6%     PYLGTCSYCHHTVPCFSPVKIEQVWDEADDNTIRIQTSAQFGYD-QSGAASANKYRYMSLKQDHTVKEGTMDDIKISTSG
 21 sp|P17517.1|POLS   99.8%  55.2%     PYXXXCADCGDGYFCYSPVAIEKIRDEASDGMLKIQVSAQIGLD-KAGTHAHTKLRYMAGHDVQESK---RDSLRVYTSA
 22 sp|P13890.1|POLS   99.8%  55.4%     PYLAXCADCGDGYFCYSPVAIEKIRDEASDGMLKIQVSAQIGLD-KAGTHAHTKMRYMAGHDVQESK---RDSLRVYTSA
 23 sp|P08491.3|POLS   99.8%  56.4%     PYLAYCADCGDGYFCYSPVAIEKIRDEAPDGMLKIQVSAQIGLD-KAGTHAHTKIRYMAGHDVQESK---RDSLRVYTSA
 24 sp|Q9JGK8.1|POLS   99.8%  53.7%     PYLAYCADCGDGQFCYSPVAIEKIRDEASDGMIKIQVAAQIGIN-KRGTHEHNKIRYIVGHYMKEAN---RDSLQVHTSG
 25 sp|Q8JJX0.1|POLS   99.3%  26.2%     PYRAYCPDC-DGTACISPIAIDEVVSSGSDHVLRMRVGSQSGVTAKGGAAGETSLRYLGRDGKVHAAD--NTRLVVRTTA
 26 sp|P03315.1|POLS   99.8%  57.2%     PYIAYCADCGAGHSCHSPVAIEAVRSEATDGMLKIQFSAQIGID-KSDNHDYTKIRYADGHAIENAV---RSSLKVATSG
 27 sp|P0DJZ6.1|POLSF  99.8%  57.2%     PYIAYCADCGAGHSCHSPVAIEAVRSEATDGMLKIQFSAQIGID-KSDNHDYTKIRYADGHAIENAV---RSSLKVATSG
 28 sp|Q8QL52.1|POLS   99.3%  25.9%     PYRAYCPDC-DGTACISPIAIDEVVSSGSDHVLRIRVGSQSGVTAKGGAAGETSLRYLGRDGKVYAAD--NTRLVVRTTA
 29 sp|P36329.1|POLS   99.3%  33.9%     PYMARCIRCAVGS-CHSPIAIEAVKSDGHDGYVRLQTSSQYGLD-SSGNLKGRTMRYDMHGTIEEIP---LHQVSLHTSR
 30 sp|P36330.1|POLS   99.3%  33.9%     PYMARCVRCAVGS-CHSPIAIEAVKSDGHDGYVRLQTSSQYGLD-PSGNLKSRTMRYNMYGTIEEIP---LHQVSLHTSR
 31 sp|P36331.1|POLS   99.3%  35.9%     PYMARCIRCAVGS-CHSPIAIEAVRSEGHDGYVRLQTSSQYGLD-PSGNLKGRTMRYDMHGTIEEIP---LHQVSLHTSR
 32 sp|P36332.1|POLS   99.3%  34.4%     PYMARCIRCAVGS-CHSPIAIEAVKSDGHDGYVRLQTSSQYGLD-SSGNLKGRTMRYDMHGTIEEIP---LHQVSLHTSR
 33 sp|P05674.1|POLS   99.3%  34.4%     PYMARCIRCAVGS-CHSPIAIEAVKSDGHDGYVRLQTSSQYGLD-SSGNLKGRTMRYDMHGTIKEIP---LHQVSLYTSR
 34 sp|P09592.2|POLS   99.3%  34.4%     PYMARCIRCAVGS-CHSPIAIEAVKSDGHDGYVRLQTSSQYGLD-SSGNLKGRTMRYDMHGTIKEIP---LHQVSLHTSR
 35 sp|P13897.1|POLS   99.3%  37.1%     PYLGFCPYCRHSAPCFSPIKIENVWDESDDGSIRIQVSAQFGYN-QAGTADVTKFRYMSFDHDHDIKEDSMDKIAISTSG
    consensus/100%                      PY...C..C.....C.SPh.l-tl.spu.st.l+hphu...Ght..ts.ht.t.hpa...t..........tth.h.oot
    consensus/90%                       PYhu.C..Ct.s..C.SPltlEtlhs-upsshl+lQhu..hGhp.pss.ht.thhpa...t...ph....httl.l.Tot
    consensus/80%                       PYhAhCspCu.up.ChSPlAIEtlhs-upDGhl+IQsS.QhGlp.pssshphsphRYhttpt.tph....hppl.ltTSu
    consensus/70%                       PYlAhCscCupGp.CaSPlAIEpl+s-ApDGhl+IQsSuQhGlp.pssshshsphRYhstcs.tpht...hsplhlpTSu
```

```
                           cov     pid  81         .         1         .         .         .         .         :         . 160
  1 sp|Q8JUX5.3|POLS   100.0% 100.0%     PCTITGTMGHFILARCPKGETLTVGFTDSRKISHSCTHPFHHDPPVIGREKFHSRPQHGKELPCSTYVQSNAATAEEIEV
  2 sp|Q5WQY5.1|POLS   100.0%  95.3%     PCTITGTMGHFILARCPKGETLTVGFTDGRKISHSCTHPFHHDPPVIGREKFHSRPQHGKELPCSTYVQSNAATAEEVEV
  3 sp|P0DOK1.1|POLSF 100.0%  99.5%     PCTITGTMGHFILARCPKGETLTVGFTDSRKISHSCTHPFHHDPPVIGREKFHSRPQHGKELPCSTYVQSNAATAEEIEV
  4 sp|Q5XXP3.1|POLS   100.0%  92.8%     PCTITGTMGHFILARCPKGETLTVGFTDSRKISHTCTHPFHHEPPVIGRERFHSRPQHGKELPCSTYVQSTAATAEEIEV
  5 sp|P22056.1|POLS   100.0%  82.7%     PCTITGTMGHFILARCPKGETLTVGFVDSRRISHTCMHPFRHEPPLIGREKFHSRPQHGKELPCSTYVHTTAATAEEIEV
  6 sp|O90371.1|POLS   100.0%  82.9%     PCTITGTMGHFILARCPKGETLTVGFVDSRRISHTCMHPFHHEPPLIGREKFHSRPQHGKELPCSTYVHTTAATTEEIEV
  7 sp|O90369.1|POLS   100.0%  82.9%     PCTITGTMGHFILARCPKGETLTVGFVDSRRISHTCMHPFHHEPPLIGREKFHSRPQHGKELPCSTYVHTTAATTEEIEV
  8 sp|Q86925.2|POLS    99.3%  35.9%     PCLHLSHKGYFVIVKCPPGDSITTSLK-VHGSDQTCTIPMRVGYKFVGREKYTLPPMHGTQIPCLTYERTREKSAGYVTM
  9 sp|P89946.2|POLS    99.3%  40.9%     PCDILATMGHFILARCRPGSQVEVSLSTDPK--LLCRTPFSHKPRFIGNEKSPAPTGHKTRIPCKTYSHQTDLTREEITM
 10 sp|P08768.1|POLS    98.8%  38.7%     PCSLVSHHGYYILAQCPPGDTVTVGFH-DGPNRHTCRLAHKVEFRPVGREKYRHPPEHGVELPCNRYTHKRADQGHYVEM
 11 sp|Q4QXJ7.1|POLS    98.8%  39.7%     PCSLVSHHGYYILAQCPPGDTVTVGFH-DGPNRHTCTVAHKVEFRPVGREKYRHPPEHGVELPCNRYTHKRADQGHYVEM
 12 sp|Q306W5.1|POLS    98.8%  37.7%     PCSLVSYHGYYLLAQCPPGDTVTVGFQ-DGANKHMCTIAHKVEFRPVGREKYRHPPAHGVELPCNKYTHKRADQGHYVEM
 13 sp|Q306W7.1|POLS    98.8%  38.4%     PCSLVSYHGYYILAQCPPGDTVTVGFQ-DGAIKHMCTIAHKVEFKPVGREKYRHPPEHGVELPCTKYTHKRADQGHYVEM
 14 sp|P27284.1|POLS    98.8%  39.7%     PCSLVSHHGYYILAQCPPGDTVTVGFH-DGPNRHTCTVAHKVEFRPVGREKYRHPPEHGVELPCNRYTHKRADQGHYVEM
 15 sp|Q5Y388.1|POLS    99.8%  53.7%     VCAIRGTMGHFIVAYCPPGDELKVQFQDAESHTQACKVQYKHAPAPVGREKFTVRPHFGIEVPCTTYQLTTAPTEEEIDM
 16 sp|Q8QZ72.1|POLS    99.8%  55.4%     ECTVTGTMGHFILAKCPPGERISVSFVDSKNEHRTCRIAYHHEQRLIGRERFTVRPHHGIELPCTTYQLTTAETSEEIDM
 17 sp|Q80S27.1|POLS    99.5%  51.0%     ECTVHGTMGHFIVATCPEGDSLTVAFVDKHKVRHACRIAYKHRVPVLGREHFTVRPHHGVELPCTTYAMRTSVTTEEIEM
 18 sp|P27285.1|POLS    99.3%  36.3%     PCRRLSYKGYFLLAKCPPGDSVTVSIA-SSNSATSCTMARKIKPKFVGREKYDLPPVHGKKIPCTVYDRLKETTAGYITM
 19 sp|P03316.1|POLS    99.3%  36.6%     PCRRLSYKGYFLLAKCPPGDSVTVSIV-SSNSATSCTLARKIKPKFVGREKYDLPPVHGKKIPCTVYDRLKETTAGYITM
 20 sp|P0DOK0.1|POLSF  99.3%  36.6%     PCRRLSYKGYFLLAKCPPGDSVTVSIV-SSNSATSCTLARKIKPKFVGREKYDLPPVHGKKIPCTVYDRLKETTAGYITM
 21 sp|P17517.1|POLS    99.8%  55.2%     ACSIHGTMGHFIVAHCPPGDYLKVSFEDADSHVKACKVQYKHNPLPVGREKFVVRPHFGVELPCTSYQLTTAPTDEEIDM
 22 sp|P13890.1|POLS    99.8%  55.4%     ACSIHGTMGHFIVAHCPPGDYLKXSFEDANSHVKACKVQYKHDPLPVGREKFVVRPHFGVELPCTSYQLTTAPTDEEIDM
 23 sp|P08491.3|POLS    99.8%  56.4%     ACSIHGTMGHFIVAHCPPGDYLKVSFEDADSHVKACKVQYKHDPLPVGREKFVVRPHFGVELPCTSYQLTTAPTDEEIDM
 24 sp|Q9JGK8.1|POLS    99.8%  53.7%     VCAIRGTMGHFIVAYCPPGDELKVQFQDAESHTQACKVQYKHDPAPVGREKFTVRPHFGIEVPCTTYQLTTAPTEEEIDM
 25 sp|Q8JJX0.1|POLS    99.3%  26.2%     KCDVLQATGHYILANCPVGQSLTVAATLDGTRHQCTTVFEHQVTEKFTRERSKGHHLSDMTKKCTRFSTTPKKSALYLVD
 26 sp|P03315.1|POLS    99.8%  57.2%     DCFVHGTMGHFILAKCPPGEFLQVSIQDTRNAVRACRIQYHHDPQPVGREKFTIRPHYGKEIPCTTYQQTTAETVEEIDM
 27 sp|P0DJZ6.1|POLSF  99.8%  57.2%     DCFVHGTMGHFILAKCPPGEFLQVSIQDTRNAVRACRIQYHHDPQPVGREKFTIRPHYGKEIPCTTYQQTTAETVEEIDM
 28 sp|Q8QL52.1|POLS    99.3%  25.9%     KCDVLQATGHYILANCPVGQSLTVAATLDGTRHQCTTVFEHQVTEKFTRERSKGHHLSDLTKKCTRFSTTPKKSALYLVD
 29 sp|P36329.1|POLS    99.3%  33.9%     PCHIVDGHGYFLLARCPAGDSITMEFK-KDAVTHSCSVPYEVKFNPVGRELYTHPPEHGAEQACQVYAHDAQNRGAYVEM
 30 sp|P36330.1|POLS    99.3%  33.9%     PCHIVDGHGYFLLARCPAGDSITMEFK-KDSVTHSCSVPYEVKFNPVGRELYTHPPEHGAEQACQVYAHDAQNRGAYVEM
 31 sp|P36331.1|POLS    99.3%  35.9%     PCHIIDGHGYFLLARCPAGDSITMEFK-KESVTHSCSVPYEVKFNPVGRELYTHPPEHGAEQPCHVYAHDAQNRGAYVEM
 32 sp|P36332.1|POLS    99.3%  34.4%     PCHIVDGHGYFLLARCPAGDSITMEFK-KGSVTHSCSVPYEVKFNPVGRELYTHPPEHGAEQACQVYAHDAQNRGAYVEM
 33 sp|P05674.1|POLS    99.3%  34.4%     PCHIVDGHGYFLLARCPAGDSITMEFK-KDSVRHSCSVPYEVKFNPVGRELYTHPPEHGVEQACQVYAHDAQNRGAYVEM
 34 sp|P09592.2|POLS    99.3%  34.4%     PCHIVDGHGYFLLARCPAGDSITMEFK-KDSVTHSCSVPYEVKFNPVGRELYTHPPEHGVEQACQVYAHDAQNRGAYVEM
 35 sp|P13897.1|POLS    99.3%  37.1%     PCRRLGHKGYFLLAQCPPGDSVTVSIT-SGASENSCTVEKKIRRKFVGREEYLFPPVHGKLVKCHVYDHLKETSAGYITM
    consensus/100%                       .C.hhthhGaalls.C..Gp.lp.th.........s.h..p.....hspE........th...C..a....t.p...l..
    consensus/90%                        .CthhshhGaallApCP.G-.lphth..ttt..psCph.hcht...lGREha...P.aGhp.sCphY...tt.pst.lph
    consensus/80%                        sCplhsthGaFlLApCP.G-plpstht.stt.tpsCph.a+hc...lGRE+aph.PtaGhchsCpsYtppttspst.lph
    consensus/70%                        sCplhushGaFlLApCPsG-olTVuFp.sps.p+sCplsa+hc...lGRE+aph.PpHGhElPCssYsppstspst.l-M

                           cov     pid 161         .         .         .         2         .         .         .         . 240
  1 sp|Q8JUX5.3|POLS   100.0% 100.0%     HM---PPDTPDRTLLSQQSGNVKITV-NGRTVRYKCNCGG-SNEGLITTDKVINNCKVD-QCHAAVTNHKKWQYNSPLVP
  2 sp|Q5WQY5.1|POLS   100.0%  95.3%     HM---PPDTPDRTLMSQQSGNVKITV-NSQTVRYKCNCGD-SNEGLTTTDKVINNCKVD-QCHAAVTNHKKWQYNSPLVP
  3 sp|P0DOK1.1|POLSF 100.0%  99.5%     HM---PPDTPDRTLLSQQSGNVKITV-NSQTVRYKCNCGG-SNEGLITTDKVINNCKVD-QCHAAVTNHKKWQYNSPLVP
  4 sp|Q5XXP3.1|POLS   100.0%  92.8%     HM---PPDTPDRTLMTQQSGNVKITV-NGQTVRYKCNCGG-SNEGLTTTDKVINNCKID-QCHAAVTNHKNWQYNSPLVP
  5 sp|P22056.1|POLS   100.0%  82.7%     HM---PPDTPDYTLMTQQAGNVKITV-DGQTVRYKCKCDG-SNEGLITADKVINNCKVD-QCHTAVTNHKKWQYNSPLTP
```

```
  6 sp|O90371.1|POLS  100.0%  82.9%    HM---PPDTPDYTLMTQQAGNVKITV-DGQTVRYKCKCDG-SNEGLITTDKVINNCKVD-QCHTAVTNHKKWQYNSPLTP
  7 sp|O90369.1|POLS  100.0%  82.9%    HM---PPDTPDYTLMTQQAGNVKITV-DGQTVRYKCKCDG-SNEGLITTDKVINNCKVD-QCHTAVTNHKKWQYNSPLTP
  8 sp|Q86925.2|POLS   99.3%  35.9%    HR---PGQQSITMLMEESGGEVYVQPTSGRNVTYECKCG-DFKTGTVTARTKIDGCTERKQCIAISADHVKWVFNSPDLI
  9 sp|P89946.2|POLS   99.3%  40.9%    HV---PPDVPIQGLVSNTGKSYSLDP-KTKTIKYKCTCGETVKEGTATNKITLFNCDTAPKCITYAVDNTVWQYNSQYVP
 10 sp|P08768.1|POLS   98.8%  38.7%    HQ---PGLVGDHSLLSIHSAKVKITVPSGAQVKYYCKCP--DVREGITSSDHTTTCTDVKQCRAYLIDNKKWVYNSGRLP
 11 sp|Q4QXJ7.1|POLS   98.8%  39.7%    HQ---PGLVADHSLLSIHSAKVKITVPSGAQVKYYCKCP--DVREGITSSDHTTTCTDVKQCRAYLIDNKKWVYNSGRLP
 12 sp|Q306W5.1|POLS   98.8%  37.7%    HQ---PGLVADHSLLSLSSTKVKITVPSGSQVKYYCKCP--DVQEGTTSGDHTTTCTDLKQCRAYLIDNKKWVFNSGKLP
 13 sp|Q306W7.1|POLS   98.8%  38.4%    HQ---PGLVADHSLLSMSSTKVKITVPSGSQVKYYCKCP--DVKEGTTGSDYTTACTDLKQCRAYLIDNKKWVYNSGKLP
 14 sp|P27284.1|POLS   98.8%  39.7%    HQ---PGLVADHSLLSIHSAKVKITVPSGAQVKYYCKCP--DVREGITSSDHTTTCTDVKQCRAYLIGNKKWVYNSGRLP
 15 sp|Q5Y388.1|POLS   99.8%  53.7%    HT---PPDIPDITLLSQQSGNVKITA-GGKTIRYNCTCGS-GNVGTTSSDKTINSCKIA-QCHAAVTNHDKWQYTSSFVP
 16 sp|Q8QZ72.1|POLS   99.8%  55.4%    HM---PPDIPDRTILSQQSGNVKITV-NGRTVRYSSSCGS-QAVGTTTTDKTINSCTVD-KCQAYVTSHTKWQFNSPFVP
 17 sp|Q80S27.1|POLS   99.5%  51.0%    HV---AHDVPDNTFLSKTGNKVKITP-KGKSIRYNCTCGS-KESGVTKQDKEFDNCEVS-QCHTMVTAHDKWQFNSPYVP
 18 sp|P27285.1|POLS   99.3%  36.3%    HR---PGPHAYTSYLEESSGKVYAKPPSGKNITYECKCG-DYKTGTVTTRTEITGCTAIKQCVAYKSDQTKWVFNSPDLI
 19 sp|P03316.1|POLS   99.3%  36.6%    HR---PRPHAYTSYLEESSGKVYAKPPSGKNITYECKCG-DYKTGTVSTRTEITGCTAIKQCVAYKSDQTKWVFNSPDLI
 20 sp|P0DOK0.1|POLSF  99.3%  36.6%    HR---PRPHAYTSYLEESSGKVYAKPPSGKNITYECKCG-DYKTGTVSTRTEITGCTAIKQCVAYKSDQTKWVFNSPDLI
 21 sp|P17517.1|POLS   99.8%  55.2%    HT---PPDIPDRTLLSQTAGNVKITA-GGRTIRYNCTWGR-DNVGTTSTDKTINACKID-QCHAAVTSHDKWQFTSPFVP
 22 sp|P13890.1|POLS   99.8%  55.4%    HT---PPDIPDRTLLSQTAGNVKITA-GGRTIRYNCTCGR-DNVGTTSTDKTINTCKID-QCHAAVTSHDKWXFTSPFVP
 23 sp|P08491.3|POLS   99.8%  56.4%    HT---PPDIPDRTLLSQTAGNVKITA-GGRTIRYNCTCGR-DNVGTTSTDKTINTCKID-QCHAAVTSHDKWQFTSPFVP
 24 sp|Q9JGK8.1|POLS   99.8%  53.7%    HT---PPDIPDITLLSQQSGDVKITA-GGKTIRYNCTCGS-GNVGTTSSDKTINSCKIA-QCHAAVTNHDKWQYTSSFVP
 25 sp|Q8JJX0.1|POLS   99.3%  26.2%    VYDALPISVEISTVVTCSDSQCTVRVPPGTTVKFDKKCKSADSATVTFTSDSQTFTCEEPVLTAASITQGKPHLRSAMLP
 26 sp|P03315.1|POLS   99.8%  57.2%    HM---PPDTPDRTLLSQQSGNVKITV-GGKKVKYNCTCGT-GNVGTTNSDMTINTCLIE-QCHVSVTDHKKWQFNSPFVP
 27 sp|P0DJZ6.1|POLSF  99.8%  57.2%    HM---PPDTPDRTLLSQQSGNVKITV-GGKKVKYNCTCGT-GNVGTTNSDMTINTCLIE-QCHVSVTDHKKWQFNSPFVP
 28 sp|Q8QL52.1|POLS   99.3%  25.9%    VYDALPTSVEISTVVTCNERQCTVRVPPGTTVKFDKRCKNAAKETVTFTSDSQTFTCEEPVLTAASITQGKPHLRSSMLP
 29 sp|P36329.1|POLS   99.3%  33.9%    HL---PGSEVDSSLVSLSGSSVTVTPPAGTSALVECECGGTKISETINTAKQFSQCTKKEQCRAYRLQNDKWVYNSDKLP
 30 sp|P36330.1|POLS   99.3%  33.9%    HL---PGSEVDSSLVSLSSGLVSVTPPAGTSALVECECSGTTISKTINKTKQFSQCTKKEQCRAYRLQNDKWVYNSDKLP
 31 sp|P36331.1|POLS   99.3%  35.9%    HL---PGSEVDSTLLSTSGSSVHVTPPAGQSVLVECECGGTKISETINSAKQYSQCSKTAQCRAYRTQNDKWVYNSDKLP
 32 sp|P36332.1|POLS   99.3%  34.4%    HL---PGSEVDSSLISLSGSSVTVTPPVGTSALVKCKCGGTKISETINKAKQFSQCTKKEQCRAYRLQNDKWVYNSDKLP
 33 sp|P05674.1|POLS   99.3%  34.4%    HL---PGSEVDSSLVSLSGSSVTVTPPDGTSALVECECGGTKISETINKTKQFSQCTKKEQCRAYRLQNDKWVYNSDKLP
 34 sp|P09592.2|POLS   99.3%  34.4%    HL---PGSEVDSSLVSLSGSSVTVTPPVGTSALVECECGGTKISETINKTKQFSQCTKKEQCRAYRLQNDKWVYNSDKLP
 35 sp|P13897.1|POLS   99.3%  37.1%    HR---PGPHAYKSYLEEASGEVYIKPPSGKNVTYECKCG-DYSTGIVSTRTKMNGCTKAKQCIAYKSDQTKWVFNSPDLI
    consensus/100%                     h....s.......hhp.ttt.h.hps..stphhh.pph......thh.tt.....s.....h.s...tp....hpS..h.
    consensus/90%                      H....P...s..ohhp.puspVhlps.sGpphhh.CpCs.....thhsptp.hstCp...pChshh.pppKW.asSs.l.
    consensus/80%                      Hh...Psshs.pollo.puupVplss.sGpslpapCpCs..t..thhsssp.hstCp...QCpAhhhsppKW.aNSshlP
    consensus/70%                      Hh...PsshsDpoLloppuupVplTs.sGpslpYpCpCut.t.ptshsoscphssCpht.QC+AhhhspcKW.aNSshlP

                        cov    pid 241         :         .         .         .         .         3         .         . 320
  1 sp|Q8JUX5.3|POLS  100.0% 100.0%    RNAELGDRKGKIHIPFPLANVTCMVPKARNPTVTYGKNQVIMLLYPDHPTLLSYRSMGEEPNYQEEWVTHKKEVVLTVPT
  2 sp|Q5WQY5.1|POLS  100.0%  95.3%    RNVELGDRKGKIHIPFPLANVTCRGPKARNPTVTYGKNQVIMLLYPDHPTLLSYRNMGEEPNYQEEWVTHKKEVRLTVPT
  3 sp|P0DOK1.1|POLSF 100.0%  99.5%    RNAELGDRKGKIHIPFPLANVTCMVPKARNPTVTYGKNQVIMLLYPDHPTLLSYRSMGEEPNYQEEWVTHKKEVVLTVPT
  4 sp|Q5XXP3.1|POLS  100.0%  92.8%    RNAELGDRKGKIHIPFPLANVTCRVPKARNPTVTYGKNQVTMLLYPDHPTLLSYRNMGQEPNYHEEWVTHKKEVTLTVPT
  5 sp|P22056.1|POLS  100.0%  82.7%    RNSEQGDRKGKIHIPFPLVNTTCRVPKARNPTVTYGKNRVTLLLHPDHPTLLSYRAMGRIPDYHEEWITNKKEISITVPA
  6 sp|O90371.1|POLS  100.0%  82.9%    RNSEQGDRKGKIHIPFPLVNTTCRVPKARNPTVTYGKNRVTLLLYPDHPTLLSYRAMGRIPDYHEEWITSKKEISITVPA
  7 sp|O90369.1|POLS  100.0%  82.9%    RNSEQGDRKGKIHIPFPLVNTTCRVPKARNPTITYGKNRVTLLLYPDHPTLLSYRSMGRIPDYHEEWITSKKEISITVPA
  8 sp|Q86925.2|POLS   99.3%  35.9%    R-HTDHTAQGKLHIPFPLQQAQCTVPLAHLPGVKHAYRSMSLTLHAEHPTLLTTRHLGENPQPTAEWIVGSVTRNFSITI
  9 sp|P89946.2|POLS   99.3%  40.9%    R-SEVTEVKGKIHVPFPLTDSTCAVSVAPEPQVTYRLGEVEFHFHPMYPTLFSIRSLGKDPSHSQEWIDTPMSKTIQVGA
 10 sp|P08768.1|POLS   98.8%  38.7%    R-GEGDTFKGKLHVPFVPVKAKCIATLAPEPLVEHKHRTLILHLHPDHPTLLTTRSLGSDANPTRQWIERPTTVNFTVTG
 11 sp|Q4QXJ7.1|POLS   98.8%  39.7%    R-GEGDTFKGKLHVPFVPVKAKCIATLAPEPLVEHKHRTLILHLHPDHPTLLTTRSLGSDANPTRQWIERPTTVNFTVTG
```

```
12 sp|Q306W5.1|POLS    98.8%  37.7%     R-GEGETFKGKLHVPFVPVTSKCTATLAPEPLVEHKHRSLILHLHPEHPTLLTTRALGNDARPTRQWVDQPTTVNFTVTG
13 sp|Q306W7.1|POLS    98.8%  38.4%     R-GEGETFKGKLHVPFVPVTSKCTATLAPEPLVEHKHRSLVLHLHPEHPTLLTTRALGSNARPTRQWIEQPTTVNFTVTG
14 sp|P27284.1|POLS    98.8%  39.7%     R-GEGDTFKGKLHVPFVPVKAKCIATLAPEPLVEHKHRTLILHLHPDHPTLLTTRSLGSDANPTRQWIERPTTVNFTVTG
15 sp|Q5Y388.1|POLS    99.8%  53.7%     R-ADQLSRKGKVHVPFPLTNSTCRVPVARAPGVTYGKRELTVKLHPDHPTLLTYRSLGADPRPYEEWIDRYVERTIPVTE
16 sp|Q8QZ72.1|POLS    99.8%  55.4%     R-RMQAERKGKVHIPFPLINTTCRVPLAPEALVRSGKREATLSLHPIHPTLLSYRTFGAERVFDEQWITAQTEVTIPVPV
17 sp|Q80S27.1|POLS    99.5%  51.0%     R--AGSGKKGKIHVPFPLSNSTCRVPLAPLPNTIPAKNGITLQLHPVAPTLLTYRTLGEKPEHHTEWISESCERTLPVPE
18 sp|P27285.1|POLS    99.3%  36.3%     R-HADHTAQGKLHLPFKLIPSTCMVPVAHAPNVIHGFKHISLQLDTDHLTLLTTRRLGANPEPTTEWIIGKTVRNFTVDR
19 sp|P03316.1|POLS    99.3%  36.6%     R-HDDHTAQGKLHLPFKLIPSTCMVPVAHAPNVIHGFKHISLQLDTDHLTLLTTRRLGANPEPTTEWIVGKTVRNFTVDR
20 sp|P0DOK0.1|POLSF   99.3%  36.6%     R-HDDHTAQGKLHLPFKLIPSTCMVPVAHAPNVIHGFKHISLQLDTDHLTLLTTRRLGANPEPTTEWIVGKTVRNFTVDR
21 sp|P17517.1|POLS    99.8%  55.2%     R-ADQTARKGKVHVPFPLTNVTCRVPLARAPDVTYGKKEVTLRLHPDHPTLFSYRSLGAEPHPYEEWVDKFSERIIPVTE
22 sp|P13890.1|POLS    99.8%  55.4%     R-ADQTARKGKVHVPFPLTNVTCRVPLARAPDVTYGKKEVTLRLHPDHPTXFSYRSLGAVPHPYEEWVDKFSERIIPVTE
23 sp|P08491.3|POLS    99.8%  56.4%     R-ADQTARRGKVHVPFPLTNVTCRVPLARAPDVTYGKKEVTLRLHPDHPTLFSYRSLGAEPHPYEEWVDKFSERIIPVTE
24 sp|Q9JGK8.1|POLS    99.8%  53.7%     R-ADQLPRKGKVHVPFPLTNSTCRVPLARAPGVTYGKRELTVKLHPDHPTLLTYRSLGADPRPYEEWIDRYVERTIPVTE
25 sp|Q8JJX0.1|POLS    99.3%  26.2%     S--GGKEVKARIPFPFPPETATCRVSVAPLPSITYEESDVLLAGTAKYPVLLTTRNLGFHSNATSEWIQGKYLRRIPVTP
26 sp|P03315.1|POLS    99.8%  57.2%     R-ADEPARKGKVHIPFPLDNITCRVPMAREPTVIHGKREVTLHLHPDHPTLFSYRTLGEDPQYHEEWVTAAVERTIPVPV
27 sp|P0DJZ6.1|POLSF   99.8%  57.2%     R-ADEPARKGKVHIPFPLDNITCRVPMAREPTVIHGKREVTLHLHPDHPTLFSYRTLGEDPQYHEEWVTAAVERTIPVPV
28 sp|Q8QL52.1|POLS    99.3%  25.9%     S--GGKEVKARIPFPFPPETATCRVSIAPLPSITYEESDVLLAGTAKYPVLLTTRNLGFHSNATSEWIQGKYLRRIPVTP
29 sp|P36329.1|POLS    99.3%  33.9%     K-AAGATLKGKLHVPFLLADGKCTVPLAPEPMITFGFRSVSLKLHPKNPTYLTTRQLADEPHYTHELISEPVVRNFSVTE
30 sp|P36330.1|POLS    99.3%  33.9%     K-AAGATLKGKLHVPFLLADGKCTVPLAPEPMITFGFRSVSLKLHPKYPTYLTTRELADEPHYTHELISEPSVRNFSVTA
31 sp|P36331.1|POLS    99.3%  35.9%     K-AAGETLKGKLHVPFVLTEAKCTVPLAPEPIITFGFRSVSLKLHPKNPTFLTTRQLDGEPAYTHELITNPVVRNFSVTE
32 sp|P36332.1|POLS    99.3%  34.4%     K-AAGATLKGKLHVPFLLADGKCTVPLAPEPMITFGFRSVSLKLHPKNPTYLTTRQLADEPHYTHELISEPAVRNFTVTE
33 sp|P05674.1|POLS    99.3%  34.4%     K-AAGATLKGKLHVPFLLADGKCTVPLAPEPMITFGFRSVSLKLHPKNPTYLITRQLADEPHYTHELISEPAVRNFTVTE
34 sp|P09592.2|POLS    99.3%  34.4%     K-AAGATLKGKLHVPFLLADGKCTVPLAPEPMITFGFRSVSLKLHPKNPTYLTTRQLADEPHYTHELISEPAVRNFTVTE
35 sp|P13897.1|POLS    99.3%  37.1%     R-HTDHSVQGKLHIPFRLTPTVCPVPLAHTPTVTKWFKGITLHLTATRPTLLTTRKLGLRADATAEWITGTTSRNFSVGR
   consensus/100%                       p......hpu+l.hPF......C.sshA..s.h....tth.h.h.s...s.h.hRths......tphl.t...h.h.ls.
   consensus/90%                        +..t..thpGKlHlPF....spChsshA..P.l.athppl.h.Lhsp.PThhohRthut.sp..pphl.t...h.hsVs.
   consensus/80%                        +.ttttshKGKlHlPF.LssspChVshA.tP.lpathppl.LtLaPcaPTLLohRphGtpsp.ppEWlst.h.hshsVst
   consensus/70%                        R.utttshKGKlHlPFsLssspCpVPlA.tPhVpauhpplsLpLaPc+PTLLohRpLGtcPp.pcEWIsp.s.hshsVst

                      cov     pid 321          .       .       :       .       .       .       .      4 400
 1 sp|Q8JUX5.3|POLS   100.0% 100.0%     EGLEVTWGNNEPYKYWPQL-SANGTAHGHPHEIILYYYELYPTMTVVVVSVASFILLSMVGMAVGMCMCARRRCITPYEL
 2 sp|Q5WQY5.1|POLS   100.0%  95.3%     EGLEVTWGNNEPYKYWPQL-STNGTAHGHPHEIILYYYELYPTMTVVVVSVASFVLLSMVGVAVGMCMCARRRCITPYEL
 3 sp|P0DOK1.1|POLSF  100.0%  99.5%     EGLEVTWGNNEPYKYWPQL-SANGTAHGHPHEIILYYYELYPTMTVVVVSVASFILLSMVGMAVGMCMCARRRCITPYEL
 4 sp|Q5XXP3.1|POLS   100.0%  92.8%     EGLEVTWGNNEPYKYWPQM-STNGTAHGHPHEIILYYYELYPTMTVVIVSVASFVLLSMVGTAVGMCVCARRRCITPYEL
 5 sp|P22056.1|POLS   100.0%  82.7%     EGLEVTWGNNDPYKYWPQL-STNGTAHGHPHEIILYYYELYPTTTIAVLAAASIVITSLVGLSLGMCICARRRCITPYEL
 6 sp|O90371.1|POLS   100.0%  82.9%     EGLEVTWGNNDPYKYWPQL-STNGTAHGHPHEIILYYYELYPTTTIAVLAAASIVVASLVGLSLGMCICARRRCITPYEL
 7 sp|O90369.1|POLS   100.0%  82.9%     EGLEVTWGNNDPYKYWPQL-STNGTAHGHPHEIILYYYELYPTTTIAVLAAASIVVASLVSLSLGMCICARRRCITPYEL
 8 sp|Q86925.2|POLS    99.3%  35.9%     QGFEYTWGNQKPVRVYAQE-SAPGNPHGWPHEIVRHYYHLYPFYTVTVLSGMGLAICAGLVISILCCCKARRDCLTPYQL
 9 sp|P89946.2|POLS    99.3%  40.9%     EGVEYVWGNNNPVRLWAQK-SSSSSAHGNPISIVSHYYDLYPYWTITVLASLGLLIVISSGFSCFLCSVARTKCLTPYQL
10 sp|P08768.1|POLS    98.8%  38.7%     EGLEYTWGNHPPKRVWAQE-SGEGNPHGWPHVVVVYYYNRYPLTTIIGLCTCVAIIMVSCDHPCGSFSGLRNLCITPYKL
11 sp|Q4QXJ7.1|POLS    98.8%  39.7%     EGLEYTWGNHPPKRVWAQE-SGEGNPHGWPHEVVVYYYNRYPLTTIIGLCTCVAIIMVSCVTSVWLLCRTRNLCITPYKL
12 sp|Q306W5.1|POLS    98.8%  37.7%     EGFEYTWGNHPPKRVWAQE-SGEGNPHGWPHEVVIYYYNRYPMTTIVGLCTCAAIIMVSCITSVWLLCRTRNLCITPYRL
13 sp|Q306W7.1|POLS    98.8%  38.4%     EGFEYTWGNHPPKRVWAQE-SGEGNPHGWPHEIVIYYYNRYPMTTVIGLCTCVAIIMVSCVTSVWLLCRTRNLCITPYRL
14 sp|P27284.1|POLS    98.8%  39.7%     EGLEYTWGNHPPKRVWAQE-SGEGNPHGWPHEVVVYYYNRYPLTTIIGLCTCVAIIMVSCVHPCGSFAGLRNLCITPYKL
15 sp|Q5Y388.1|POLS    99.8%  53.7%     DGIEYRWGNNPPVRLWAQL-TTEGKPHGWPHEIILYYYGLYPAATIAAVSAAGLAVVLSLLASCYMFATARRKCLTPYAL
16 sp|Q8QZ72.1|POLS    99.8%  55.4%     EGVEYQWGNHKPQRFVVAL-TTEGKAHGWPHEIIEYYYGLHPTTTIVVVIRVSVVVLLSFAASVYMCVVARTKCLTPYAL
17 sp|Q80S27.1|POLS    99.5%  51.0%     EGLEYTWGNHAPVRLWAQL-TTKGSAHGMPHEIFSYYYGLYPATTVAVCVGLACVILLALSASCCLCVSARNKCLTPYAL
```

```
18 sp|P27285.1|POLS    99.3%  36.3%    DGLEYIWGNHEPVRVYAQE-SAPGDPHGWPHEIVQHYYHRHPVYTILAVASAAVAMMIGVTVAALCACKARRECLTPYAL
19 sp|P03316.1|POLS    99.3%  36.6%    DGLEYIWGNHEPVRVYAQE-SAPGDPHGWPHEIVQHYYHRHPVYTILAVASATVAMMIGVTVAVLCACKARRECLTPYAL
20 sp|P0DOK0.1|POLSF   99.3%  36.6%    DGLEYIWGNHEPVRVYAQE-SAPGDPHGWPHEIVQHYYHRHPVYTILAVASATVAMMIGVTVAVLCACKARRECLTPYAL
21 sp|P17517.1|POLS    99.8%  55.2%    EGXEYQWGNNPPVRLWAXL-TTEGKPHGWPHEIIQYYYGLYPAATIAAVSGXSLMALLTLAATCCMLATARRKCLTPYAL
22 sp|P13890.1|POLS    99.8%  55.4%    EGIEYQWGNNPPVRLWAQL-TTEGKPHGWPHEIIQYYYGLYPAATIAAVSGASLMALLTLAATCCMLATARRKCLTPYAL
23 sp|P08491.3|POLS    99.8%  56.4%    EGIEYQWGNNPPVRLWAQL-TTEGKPHGWPHEIIQYYYGLYPAATIAAVSGASLMALLTLAATCCMLATARRKCLTPYAL
24 sp|Q9JGK8.1|POLS    99.8%  53.7%    DGIEYRWGNNPPVRLWAQL-TTEGKPHGWPHEIILYYYGLYPAATIAAVSAAGLAVVLSLLASCYMFATARRKCLTPYAL
25 sp|Q8JJX0.1|POLS    99.3%  26.2%    QGIELTWGNNAPMHFWSSVRYASGDADAYPWELLVYHTKHHPEYAWAFVGVACGLLAIAACMFACACSRVRYSLVANTFN
26 sp|P03315.1|POLS    99.8%  57.2%    DGMEYHWGNNDPVRLWSQL-TTEGKPHGWPHQIVQYYYGLYPAATVSAVVGMSLLALISIFASCYMLVAARSKCLTPYAL
27 sp|P0DJZ6.1|POLSF   99.8%  57.2%    DGMEYHWGNNDPVRLWSQL-TTEGKPHGWPHQIVQYYYGLYPAATVSAVVGMSLLALISIFASCYMLVAARSKCLTPYAL
28 sp|Q8QL52.1|POLS    99.3%  25.9%    QGIELMLGNNAPLHFWSSVRYASGDADAYPWELLVHHIKHHPEYAWAFVGVACGLLAVAACMFACACNRVRYSLLANTFN
29 sp|P36329.1|POLS    99.3%  33.9%    KGWEFVWGNHPPKRFWAQE-TAPGNPHGLPHEVITHYYHRYPMSTILGLSICAAIVTVSIAASTWLLCKSRVSCLTPYRL
30 sp|P36330.1|POLS    99.3%  33.9%    KGWEFVWGNHPPKRFWAQE-TAPGNPHGLPHEVIVHYYHRYPMSTITGLSICAAIVAVSIAASTWLLCRSRASCLTPYRL
31 sp|P36331.1|POLS    99.3%  35.9%    KGWEFVWGNHPPQRYWSQE-TAPGNPHGLPHEVITHYYHRYPMSTILGLSICAAIVTTSIAASVWLFCKSRISCLTPYRL
32 sp|P36332.1|POLS    99.3%  34.4%    KGWEFVWGNHPPKRFWAQE-TAPGNPHGLPHEVITHYYHRYPMSTILGLSICAAIVTVSVAASTWLFCKSRVSCLTPYRL
33 sp|P05674.1|POLS    99.3%  34.4%    KGWEFVWGNHPPKRFWAQE-TAPGNPHGLPHEVITHYYHRYPMSTILGLSICAAIATVSVAASTWLFCRSRVACLTPYRL
34 sp|P09592.2|POLS    99.3%  34.4%    KGWEFVWGNHPPKRFWAQE-TAPGNPHGLPHEVITHYYHRYPMSTILGLSICAAIATVSVAASTWLFCRSRVACLTPYRL
35 sp|P13897.1|POLS    99.3%  37.1%    EGLEYVWGNHEPVRVWAQE-SAPGDPHGWPHEIIIHYYHRHPVYTVIVLCGVALAILVGTASSAACIAKARRDCLTPYAL
   consensus/100%                     pG.Eh.hGNp.P.+hhs....s.upscu.Ph.lh.aahthaP..sh.hh...shhhh......hh.h...R..hlssh..
   consensus/90%                      cGhEh.WGNp.Ph+hasp..os.GpsHGhPHpll.aYYthaPh.Tlhsls.hshhhh..hhhshhhh.tsR..ClTPYtL
   consensus/80%                      cGhEhhWGNp.Ph+hWsQ..os.GpsHGhPHEll.aYYthYPhhTlhslsssshhhhhshshushhhspuRtpClTPYtL
   consensus/70%                      -GhEahWGNpsPhRhWuQ..os.GssHGaPHEll.aYYphYPhhTlhslussuhllhlulshushhhspuRppClTPYtL

                          cov    pid 401          .    ] 415
 1 sp|Q8JUX5.3|POLS   100.0% 100.0%    TPGATVPFLLSLICC
 2 sp|Q5WQY5.1|POLS   100.0%  95.3%    TPGATVPFLLSLICC
 3 sp|P0DOK1.1|POLSF  100.0%  99.5%    TPGATVPFLLSLICC
 4 sp|Q5XXP3.1|POLS   100.0%  92.8%    TPGATVPFLLSLLCC
 5 sp|P22056.1|POLS   100.0%  82.7%    TPGATIPFLLGVLCC
 6 sp|O90371.1|POLS   100.0%  82.9%    TPGATIPFLLGILCC
 7 sp|O90369.1|POLS   100.0%  82.9%    TPGATIPFLLGVLCC
 8 sp|Q86925.2|POLS    99.3%  35.9%    APNATVPFLVTLCCC
 9 sp|P89946.2|POLS    99.3%  40.9%    APGAQLPTFIALLCC
10 sp|P08768.1|POLS    98.8%  38.7%    APNAQVPILLALLCC
11 sp|Q4QXJ7.1|POLS    98.8%  39.7%    APNAQVPILLALLCC
12 sp|Q306W5.1|POLS    98.8%  37.7%    APNAQVPILLAVLCC
13 sp|Q306W7.1|POLS    98.8%  38.4%    APNAQVPILLAVLCC
14 sp|P27284.1|POLS    98.8%  39.7%    APNAQVPILLALLCC
15 sp|Q5Y388.1|POLS    99.8%  53.7%    TPGAVVPVTLGVLCC
16 sp|Q8QZ72.1|POLS    99.8%  55.4%    TPGAVVPVTIGVLCC
17 sp|Q80S27.1|POLS    99.5%  51.0%    TPGAVVPCTLSLLCC
18 sp|P27285.1|POLS    99.3%  36.3%    APNAVIPTSLALLCC
19 sp|P03316.1|POLS    99.3%  36.6%    APNAVIPTSLALLCC
20 sp|P0DOK0.1|POLSF   99.3%  36.6%    APNAVIPTSLALLCC
21 sp|P17517.1|POLS    99.8%  55.2%    TPGAVVPLTLGLXXC
22 sp|P13890.1|POLS    99.8%  55.4%    TPGAVVPLTLGLLXC
23 sp|P08491.3|POLS    99.8%  56.4%    TPGAVVPLTLGLLCC
```

```
24 sp|Q9JGK8.1|POLS   99.8%  53.7%     TPGAVVPVTLGVLCC
25 sp|Q8JJX0.1|POLS   99.3%  26.2%     SNPPPLTALTAALCC
26 sp|P03315.1|POLS   99.8%  57.2%     TPGAAVPWTLGILCC
27 sp|P0DJZ6.1|POLSF  99.8%  57.2%     TPGAAVPWTLGILCC
28 sp|Q8QL52.1|POLS   99.3%  25.9%     PNPPPLTALTAALCC
29 sp|P36329.1|POLS   99.3%  33.9%     TPNARMPLCLAVLCC
30 sp|P36330.1|POLS   99.3%  33.9%     TPNAKMPLCLAVLCC
31 sp|P36331.1|POLS   99.3%  35.9%     TPNARMPLCLAVLCC
32 sp|P36332.1|POLS   99.3%  34.4%     TPNARMPLCLAVLCC
33 sp|P05674.1|POLS   99.3%  34.4%     TPNARIPFCLAVLCC
34 sp|P09592.2|POLS   99.3%  34.4%     TPNARIPFCLAVLCC
35 sp|P13897.1|POLS   99.3%  37.1%     APNATVPTALAVLCC
   consensus/100%                      ssss.hsh.hsh..C
   consensus/90%                       sPsA.hPhhlullCC
   consensus/80%                       sPsA.lPhhLulLCC
   consensus/70%                       sPsAhlPhhLulLCC
```

MView 1.63, Copyright © 1997-2018 Nigel P. Brown
